# Alternative redox forms of ASNA-1 separate insulin signaling from tail-anchored protein targeting and cisplatin resistance in *C. elegans*

**DOI:** 10.1038/s41598-021-88085-y

**Published:** 2021-04-21

**Authors:** Dorota Raj, Ola Billing, Agnieszka Podraza-Farhanieh, Bashar Kraish, Oskar Hemmingsson, Gautam Kao, Peter Naredi

**Affiliations:** 1 Department of Surgery, Institute of Clinical Sciences, Sahlgrenska Academy, University of Gothenburg, 413 45 Gothenburg, Sweden; 2 Department of Surgical and Perioperative Sciences, Surgery, Umeå University, 901 85 Umeå, Sweden; 3 Department of Surgery, Sahlgrenska University Hospital, 413 45 Gothenburg, Sweden

**Keywords:** Endoplasmic reticulum, Caenorhabditis elegans, Disease model

## Abstract

Cisplatin is a frontline cancer therapeutic, but intrinsic or acquired resistance is common. We previously showed that cisplatin sensitivity can be achieved by inactivation of ASNA-1/TRC40 in mammalian cancer cells and in *Caenorhabditis elegans*. ASNA-1 has two more conserved functions: in promoting tail-anchored protein (TAP) targeting to the endoplasmic reticulum membrane and in promoting insulin secretion. However, the relation between its different functions has remained unknown. Here, we show that ASNA-1 exists in two redox states that promote TAP-targeting and insulin secretion separately. The reduced state is the one required for cisplatin resistance: an ASNA-1 point mutant, in which the protein preferentially was found in the oxidized state, was sensitive to cisplatin and defective for TAP targeting but had no insulin secretion defect. The same was true for mutants in *wrb-1*, which we identify as the *C. elegans* homolog of WRB, the ASNA1/TRC40 receptor. Finally, we uncover a previously unknown action of cisplatin induced reactive oxygen species: cisplatin induced ROS drives ASNA-1 into the oxidized form, and selectively prevents an ASNA-1-dependent TAP substrate from reaching the endoplasmic reticulum. Our work suggests that ASNA-1 acts as a redox-sensitive target for cisplatin cytotoxicity and that cisplatin resistance is likely mediated by ASNA-1-dependent TAP substrates. Treatments that promote an oxidizing tumor environment should be explored as possible means to combat cisplatin resistance.

## Introduction

Solid tumors consist of both dividing and non-dividing post-mitotic cells^1^, and both populations must be eliminated to reduce tumor bulk. The frontline cytotoxic chemotherapy cisplatin rapidly kills both dividing and non-dividing cells but via different mechanisms^2^. In dividing cells, cisplatin binds to DNA to cause DNA damage, cell cycle arrest, and apoptosis^3,4^. In non-dividing cells, it kills by binding to glutathione, elevating reactive oxygen species (ROS) levels that have downstream effects on a variety of cellular processes^5^. Identification of proteins that are rapidly oxidized after cisplatin exposure would provide important information on the mechanism of cisplatin-induced death in post-mitotic cells. If cytotoxicity requires the oxidation of specific proteins, their targeted oxidation could be combined with lower doses of cisplatin to achieve equivalent anti-tumor activity as high-dose single agent regimens. The longstanding limitation of cisplatin use is the eventual development of tumor resistance and significant side-effects of nephrotoxicity and ototoxicity. The use of lower but effective doses would address these important limitations.

Knockdown of ASNA1 increases the sensitivity of tumor cells to cisplatin-induced death^6^. *Caenorhabditis elegans* is a relevant system to model the cisplatin killing effect in mammalian post-mitotic cells, since *C. elegans asna-1* mutants are sensitive to cisplatin-induced death^7^. Human ASNA1/TRC40 is a functional substitute in worm mutants, further underlining the model’s similarity to human cells^8^. We have also shown that mouse and *C. elegans* homologs of ASNA1 promote insulin secretion and that targeted mouse knockouts develop type 2 diabetes^8,9^. The study of ASNA-1 function in cisplatin detoxification and insulin secretion allowed us to propose that these two functions are genetically separable^7^.

A possible molecular basis for the separation of functions emerges from extensive cellular and structural biology studies on ASNA-1 homologs in yeast, mammals, and plants^10–13^. The yeast homolog GET3 can adopt two alternative redox-sensitive forms with non-overlapping functions^11^. The best understood function is of the reduced dimeric form, which acts as part of a targeting complex to guide tail-anchored membrane proteins (TAPs) for insertion into the endoplasmic reticulum (ER) membrane^14–16^. However, GET3 can also act as a general chaperone and a holdase by adopting an oxidized tetrameric structure with internal disulfide bonds via oxidation of critical redox-sensitive cysteines^11,17^. In vitro, GET3 adopts a zinc-stabilized conformation needed for TAP targeting when these cysteines are reduced but acts as a holdase and general chaperone when they are oxidized^11^. Thus, a better understanding of how ASNA-1 works and which form of the protein should be targeted in different pathologies requires matching the disease with the molecular state before rational drug regimens can be devised.

Here we showed that worm ASNA-1 is a redox sensitive protein required for targeting a model TAP to the ER membrane, likely via the ER based receptor with which it interacts. We found that cisplatin exposure led to increased ROS levels and investigated links between cisplatin exposure, ASNA-1 oxidation, ASNA-1 dependent TAP targeting and cisplatin induced cytotoxicity. Using *C. elegans* genetics we determined that while ASNA-1 exists in two redox forms, only the reduced form is necessary to drive cisplatin detoxification and an excess of the oxidized form prevented cisplatin detoxification while permitting normal insulin signaling. Taken together, our study identifies ASNA-1 as a biologically relevant cisplatin target, separates insulin signaling and cisplatin resistance functions of ASNA-1 and demonstrates that one strategy to increase cisplatin sensitivity would be to divert ASNA-1 towards the oxidized state.

## Results

### ASNA-1 is present in two redox-sensitive states

**Figure 1:**
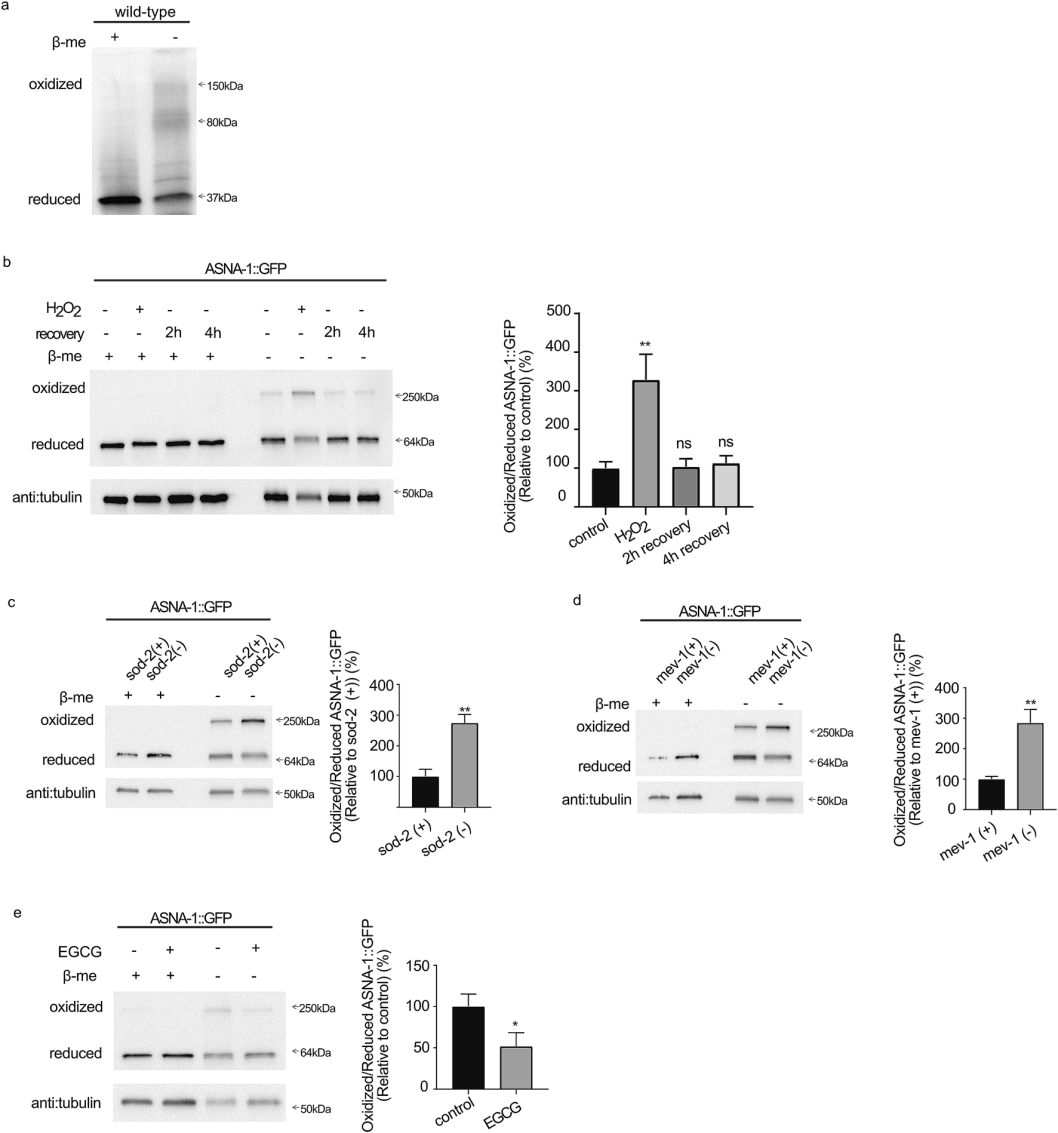
ASNA-1 is present in redox-sensitive states. (**a**) Representative image of western blot analysis following reducing and non-reducing SDS-PAGE. Non-reducing SDS-PAGE analysis allows separation of oxidized and reduced ASNA-1 in the 1-day old adult wild-type worms. The blot was probed with anti-ASNA-1 antibody. (**b**–**e**) Representative image and quantification of western blot analyses after reducing and non-reducing SDS-PAGE to detect oxidized and reduced ASNA-1::GFP in 1-day old adult animals expressing multi copy ASNA-1::GFP (b) exposed to 5 mM H_2_O_2_ for 30 min then allowed to recover for 2 or 4 h in the absence of H_2_O_2_; (**c**) *sod-2(gk257)* mutants; (**d**) *mev-1(kn1)* mutants; (**e**) exposed to the antioxidant EGCG at 5.7 µM for 48 h. Blots were probed with anti-GFP antibody and tubulin was used as a loading control. Statistical significance was determined by the independent two-sample t-test. Experiments were performed in triplicate. Bars represent mean ± SD. EGCG: Epigallocatechin gallate. For full uncropped blot source images including biological replicates used for quantification, see Supplementary Figs. S11 and S12.

The oxidation state of the *Saccharomyces cerevisiae* ASNA-1 homolog, GET3, modulates its functions. We therefore asked whether *C. elegans* ASNA-1 could also act as a redox-regulated switch and whether this switch responds to internal physiological changes. Western blot analysis following non-reducing SDS-PAGE on lysates from wild-type worms (Fig. 1a) and worms expressing ASNA-1::GFP (Fig. 1b–e), showed that *C. elegans* ASNA-1 was present in both oxidized and reduced states (Fig. 1a–e). To elucidate if the presence of GFP in ASNA-1::GFP contributes to the oxidation event of ASNA-1::GFP, we used strains expressing only GFP. GFP was not detected in the oxidized state (Supplementary Fig. S1). Substitution of conserved cysteines^11^ at positions 285 and 288 with serines prevented oxidation of ASNA-1::GFP, indicating that they are the likely redox-sensitive cysteines required for the oxidation (Supplementary Fig. S1). The ASNA-1::GFP redox balance was shifted towards more oxidized ASNA-1 (ASNA-1::GFP^OX^) at the expense of reduced ASNA-1 (ASNA-1::GFP^RED^) by exposure of worms to 5 mM H_2_O_2_. The redox balance was restored in worms removed from H_2_O_2_ (Fig. 1b), demonstrating that the changes were reversible. Consistently, the balance shifted towards more ASNA-1::GFP^OX^ at the expense of ASNA-1::GFP^RED^ in *sod-2(gk257)* and *mev-1(kn1)* mutants (Fig. 1c,d), which have high endogenous ROS levels^18^. Epigallocatechin gallate (EGCG) is an antioxidant that protects against oxidative stress and EGCG treatment of worms results in decreased ROS levels^19–21^. Levels of ASNA-1::GFP^OX^ were reduced in animals exposed to EGCG (Fig. 1e). We conclude that ASNA-1 exists in two alternative redox states, where formation of the oxidized state requires two conserved cysteines. The oxidation is reversible and can be altered in either direction by pro- and anti-oxidants and in mutants.

### ASNA-1 promotes tail-anchored protein insertion independently of its role in insulin secretion

**Figure 2:**
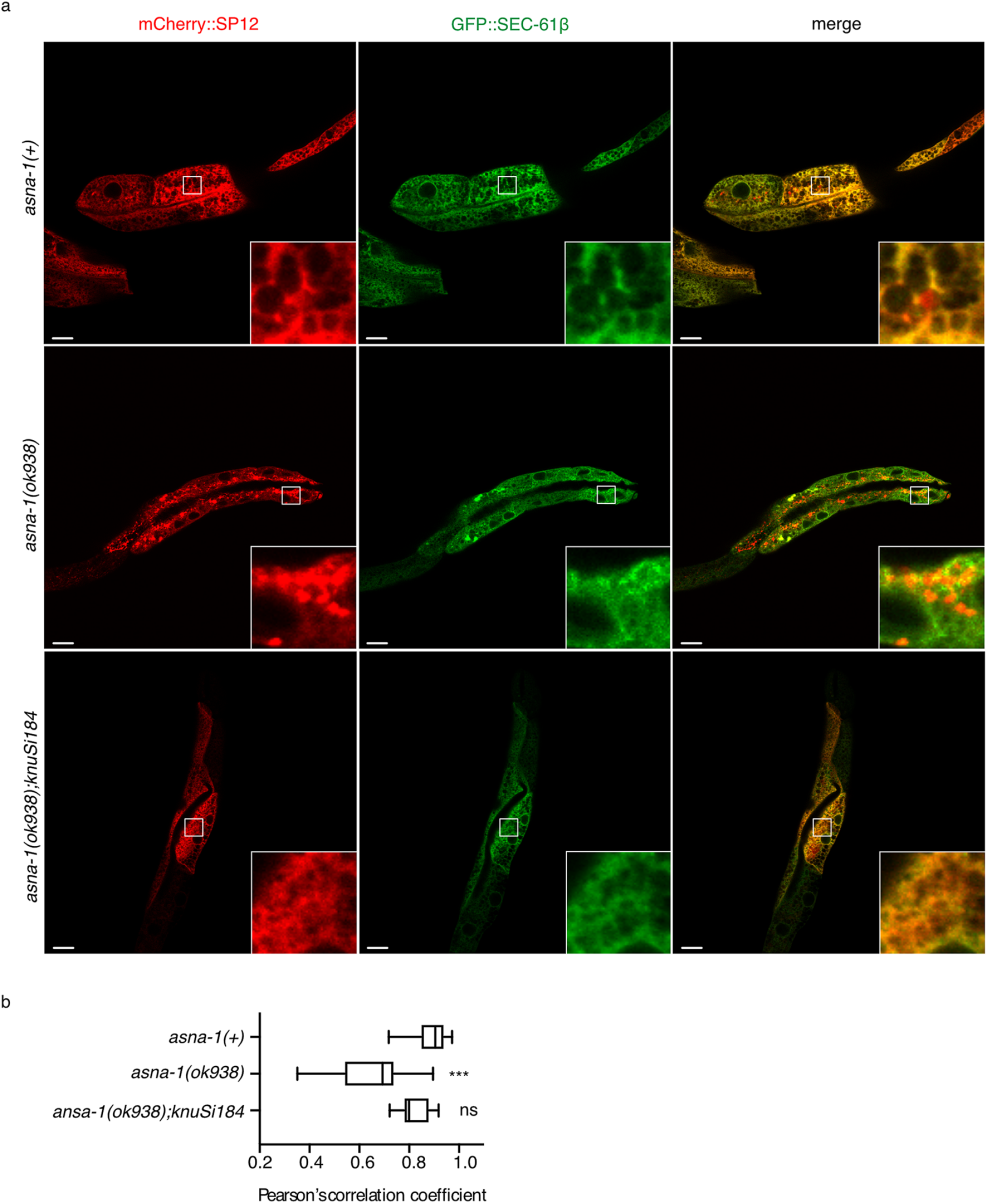
*Caenorhabditis elegans* ASNA-1 is required for the ER targeting of the TAP SEC-61β. (**a**) Representative confocal image of *asna-1(*+*)*, *asna-1(ok938)* and *asna-1(ok938);knuSi184* 1-day old adult animals co-expressing GFP::SEC-61β with mCherry::SP12 in intestinal int8 and int9 cells. Scale: 10 μm and 60 μm for magnification. (**b**) Pearson’s correlation analysis of GFP::SEC-61β and mCherry::SP12 co-localization in different strains. Box plot represents the average Pearson correlation coefficient (R) of the indicated strains. Statistical significance was determined by one-way ANOVA followed by Sidak post-hoc correction (n ≥ 10 in all cases).

We investigated whether worm ASNA-1 promoted TAP targeting, a function previously described in yeast (GET3) and mammalian (ASNA1/TRC40) homologs. We therefore established an in vivo model for TAP targeting in *C. elegans.* In vertebrates and yeast, SEC61β is a model TAP that requires ASNA1/TRC40 for correct targeting to the ER membrane^14–16,22^. Specifically, ASNA1 homologs bind to the transmembrane domain (TMD) of SEC61β. This domain is highly conserved in the *C. elegans* homolog *sec-61.B* (hereafter called SEC-61β). Indeed, using Co-IP/MS/MS analysis SEC-61β was detected as an ASNA-1::GFP-interacting partner (Supplementary Table S1). Cell fractionation using GFP-tagged SEC-61β confirmed that the protein was found only in the membrane fraction, even when expressed under a strong intestine-specific promoter (Supplementary Fig. S2). Co-expression of GFP::SEC-61β in intestinal cells of wild-type *C. elegans* with the mCherry-tagged rough ER (RER)-specific protein, SP12 (*spcs-1*), resulted in near complete colocalization in a pattern characteristic of ER (Fig. 2a,b). This colocalization pattern was disrupted in animals expressing GFP::SEC-61β^ΔTMD^ in which the TMD is deleted. Instead, large aggregates of GFP::SEC-61β^ΔTMD^ accumulated in cytoplasmic regions distinct from the RER (Supplementary Fig. S2). Insertion of the SEC-61β can be also monitored by N-linked glycosylation which can distinguish between inserted and non-inserted protein due to enzymatic activity in the ER lumen. Therefore, we constructed a C-terminally opsin-tagged SEC-61β variant (Supplementary Figure S3) as a substrate to assess insertion of this TA protein. We have used 3xFlag protein tag instead of GFP protein tag to assess the TAP insertion in vivo (Fig. 2a). Glycosylation was more easily detected with the 3xFlag protein tag, due to an easier estimation of the migration shift for this smaller protein. Substantial levels of EndoH (endoglycosidase H)-sensitive protein species were observed (Supplementary Fig. S3), demonstrating that SEC-61β is efficiently integrated into the ER membrane. Taken together, this established that worm SEC-61β was an ASNA-1-interacting partner that localized and inserted into ER membranes via its TMD and thus was a good model protein to further study the contribution of ASNA-1 to TAP targeting. This localization required ASNA-1 since, in the worm *asna-1(ok938)* protein null mutant^8^, the ER localization of GFP::SEC-61β was significantly decreased and the protein was instead detected in cytoplasmic foci (Fig. 2a,b). The total level of the SEC-61β protein was significantly decreased in absence of ASNA-1 (Supplementary Fig. S10) which led to the conclusion that SEC-61β protein is degraded when its insertion into the membrane fails. This finding was consistent with observations from other studies^23,24^. Significant reduction in glycosylation of 3xFlag::SEC-61β::opsin in *asna-1(ok938)* mutant background was not observed (Supplementary Fig. S3) probably due diminished steady-state levels of the protein. The targeting defect was solely due to lack of ASNA-1, since wild-type ASNA-1 expressed from a transgene completely rescued the TAP targeting phenotype of the mutant (Fig. 2a,b).

Electron microscopy analysis of *asna-1(ok938)* mutants revealed cells with dilated RER lumen and structures resembling proteinaceous aggregates (Supplementary Fig. S4). The ER membrane was also found in autophagosomes (Supplementary Fig. S4), an ER stress-related structure, very similar to that seen in ASNA-1-deficient mice^9^. Consistently, a transgene-based assay also revealed that autophagy levels were high in *asna-1(ok938)* mutants (Supplementary Fig. S4). The abnormal RER membranes in mutant animals were competent for proper membrane targeting of another TAP, the worm homologue of CytB5 (*cytb-5.1),* which is an ASNA1/TRC40-independent, ER-specific TAP in other systems^14,25^. GFP::CYTB5.1 localized to the ER in *asna-1(ok938)* mutants to the same extent as in wild-type animals (Supplementary Fig. S5). This was also true for another ASNA-1-independent TAP, SERP-1.1(F59F4.2) (Supplementary Fig. S5). These findings further underscored the validity of our in vivo model, since it enabled us to distinguish between ASNA-1-dependent- and independent TAP insertion.

*asna-1* mutants have multiple phenotypes, including elevated autophagy levels (Supplementary Fig. S4), high ER stress^26^, and low insulin signaling levels^7,8^. We asked whether these phenotypes contribute to the TAP targeting defect in *asna-1(ok938)* mutants. Inducing ER stress in wild-type animals to levels equivalent to those seen in *asna-1(ok938)* mutants (Supplementary Fig. S4) did not affect GFP::SEC-61β targeting (Supplementary Fig. S6). Starvation, which reduces insulin signaling and increases autophagy levels in *C. elegans*^27^ and disrupts insulin secretion in *unc-31*/CAPS^28^ mutants, did not affect GFP::SEC-61β targeting to the ER (Supplementary Fig. S6). Conversely, forcing high levels of DAF-28/insulin secretion in a *tom-1/tomosyn* mutant^29^ failed to suppress the TAP targeting defect of *asna-1(ok938)* animals (Supplementary Fig. S6). Therefore, changes in ER morphology, insulin secretion activity, autophagy, or ER stress levels do not contribute to TAP targeting defects in *asna-1* mutants. These findings support the hypothesis that the insulin secretion function of ASNA-1 and high ER stress levels do not contribute to defective TAP targeting in *asna-1* mutants, thereby providing the first evidence of independent ASNA-1 functions in *C. elegans.*

### A receptor for TAP insertion interacts with ASNA-1 and has a role in cisplatin detoxification

**Figure 3:**
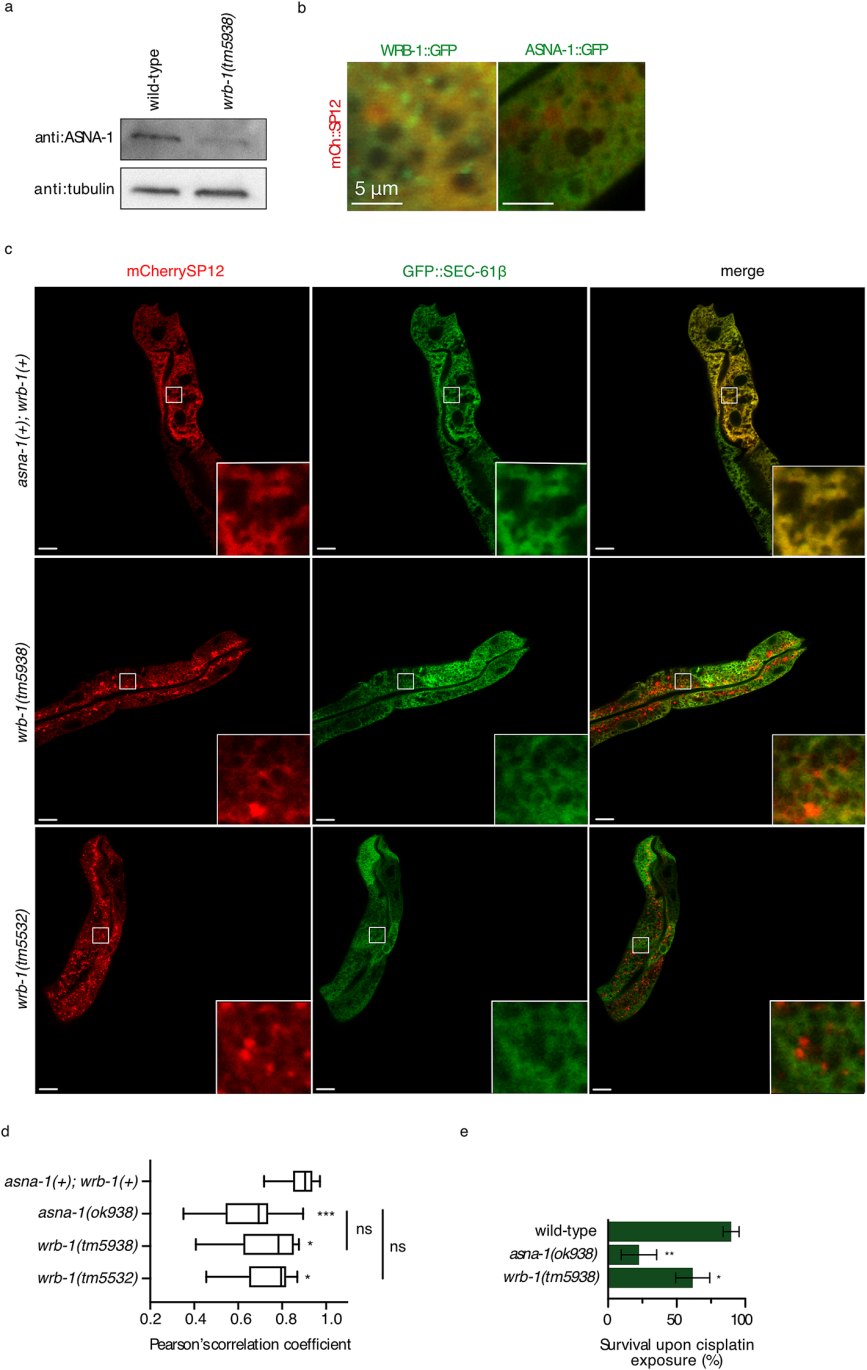
A receptor for TAP insertion has a role in cisplatin detoxification. (**a**) Western blot analysis to estimate ASNA-1 levels in wild-type and *wrb-1(tm5938)* animals. The blot was probed with an anti-ASNA-1 antibody. Tubulin was used as a loading control. For the full uncropped blot source image, see Supplementary Fig. 13. (**b**) Confocal imaging merge of animals co-expressing mCherry::SP12 with either WRB-1::GFP or ASNA-1::GFP. (**c**) Representative confocal images of *asna-1(*+*)*; *wrb-1(*+*)*, *wrb-1(tm5938)* and *wrb-1(tm5532)* 1-day old adult animals co-expressing GFP::SEC-61β with mCherry::SP12. Scale: 10 μm and 60 μm for magnification. (**d**) Pearson’s correlation analysis of GFP::SEC-61β and mCherry::SP12 co-localization in different strains. Box plot represents the average Pearson correlation coefficient (R) of the indicated strains. Statistical significance was determined by one-way ANOVA followed by Sidak post-hoc correction (n ≥ 10 in all cases). (**e**) Bars represent mean survival ± SD of 1-day-old adult animals exposed to 500 μg/mL of cisplatin for 24 h. Statistical significance was determined by the independent two-sample t-test (n ≥ 50). Survival experiments were performed in triplicate.

ASNA1/GET3-mediated insertion of TAPs requires a receptor at the ER membrane. WRB is a component of the heterodimeric receptor for TAP targeting in mammalian cells^30^. We investigated the *C. elegans* homolog WRB-1 as the possible receptor for ASNA-1. By means of Co-IP/MS/MS analysis, we detected WRB-1 as an interaction partner of ASNA-1::GFP (Supplementary Table S1). Western blot analysis revealed a decrease in ASNA-1 protein levels in *wrb-1(tm5938)* mutants (Fig. 3a), indicative of a stabilizing intracellular association. WRB-1::GFP localized to the ER (Fig. 3b) and was required for GFP::SEC-61β targeting to the ER membrane (Fig. 3c,d) to roughly the same extent as ASNA-1 (Fig. 3d). A defect in the SEC-61β insertion into the ER membrane was not detected by glycosylation assay (Supplementary Fig. S3). However, the SEC-61β steady-state protein level was decreased in *wrb-1* mutant background (Supplementary Fig. S10) to a similar extent as seen in *asna-1(ok938)* mutants indicating that degradation of SEC-61β protein occurred when it was not inserted into the ER membrane. Furthermore, *wrb-1(tm5938)* mutants displayed a phenotype similar to *asna-1* mutants for proteinaceous aggregates (Supplementary Fig. S4), swollen ER (Supplementary Fig. S4), and autophagosomes (Supplementary Fig. S4). To determine whether WRB-1 was required for other ASNA-1 functions, such as cisplatin response, we tested the survival of *wrb-1(tm5938)* mutants after exposure to cisplatin. Strikingly, *wrb-1* mutants were sensitive to cisplatin (Fig. 3e). Analysis revealed that while the life span of both mutants was short (Supplementary Fig. S7), this had no effect on cisplatin sensitivity as cisplatin provoked death was assessed in 1-day-old adults which showed no death in unexposed animals at that time point. The first natural adult death was only observed after the assay timepoint in both mutants. Thus, mutations in two different genes that similarly affect TAP targeting also demonstrated increased cisplatin sensitivity, suggesting that TAP targeting may be associated with cisplatin sensitivity.

### Cisplatin sensitivity is associated with TAP delocalization but not insulin secretion

**Figure 4:**
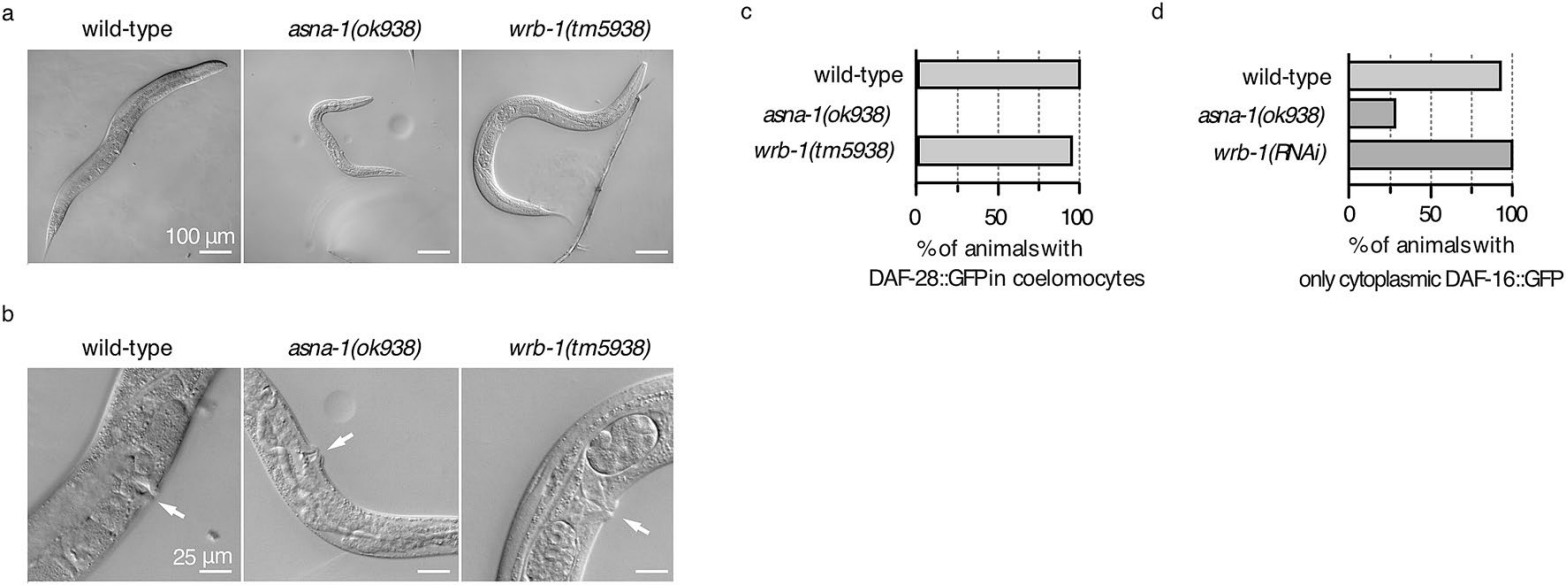
*wrb-1* mutants do not share *asna-1* insulin signaling/secretion defect. (**a**) Representative pictures of adult animals revealing differences in body size between wild type, *asna-1(ok938)*, and *wrb-1(tm5938)* strains. (**b**) Magnified image to show the germline of the animals from (**a**). White arrows indicate the vulva position for orientation purposes. (**c**) Bar graph plot represents percentage of 1-day old adult animals with secreted DAF-28::GFP in coelomocytes (n ≥ 20). (**d**) Bars graph plot represents percentage of 1-day old adult animals displaying only cytoplasmic localization of DAF-16::GFP (n ≥ 15).

We have previously shown that animals lacking maternal and zygotic *asna-1* reversibly arrest at the L1 stage with defective insulin/IGF signaling (IIS). Maternally rescued *asna-1(ok938)* mutants grow up to become pale, small, and sterile adults with severe germline defects and low levels of IIS^8^. Although both ASNA-1 and WRB-1 mutants display GFP::SEC-61β localization defects (Fig. 3c,d, Supplementary Fig. S3, Supplementary Fig. S10), *wrb-1*(*tm5938*) mutant adults were both larger and had better developed germlines (Fig. 4a,b), indicating that TAP-targeting defects may be separated from the growth and body size phenotypes that in *asna-1* mutants are associated with insulin signaling defects. To directly determine whether loss of WRB-1 affects insulin secretion, we used two transgenic worm strains reporting on IIS activity: DAF-16/FOXO::GFP^27^ and DAF-28/insulin::GFP^8^. In contrast to *asna-1(ok938)* mutants, *wrb-1(tm5938)* mutants had no DAF-28::GFP secretion defect (Fig. 4c), even though ASNA-1 protein levels were reduced (Fig. 3a). Furthermore, DAF-16/FOXO::GFP was always cytoplasmic in *wrb-1(RNAi)* animals, indicating high IIS levels (Fig. 4d). By contrast, DAF-16/FOXO:: GFP localizes to the nuclei upon *asna-1* knockdown due to insulin signaling defects^8^. Furthermore, *asna-1* and insulin receptor null mutants^8,31^ exhibit reversible first larval stage arrest. This characteristic IIS defect was not observed when both maternal and zygotic *wrb-1* gene activity (Supplementary Fig. S8) was depleted. Taken together, these results show that, compared to mutants of its physical interaction partner ASNA-1, *wrb-1* mutants showed no insulin secretion or signaling defects. These findings strongly support the conclusion that cisplatin sensitivity and TAP targeting defects are unlinked to insulin secretion defects.

### Altered redox balance that favors high ASNA-1^OX^ levels causes cisplatin sensitivity and TAP targeting defects

**Figure 5:**
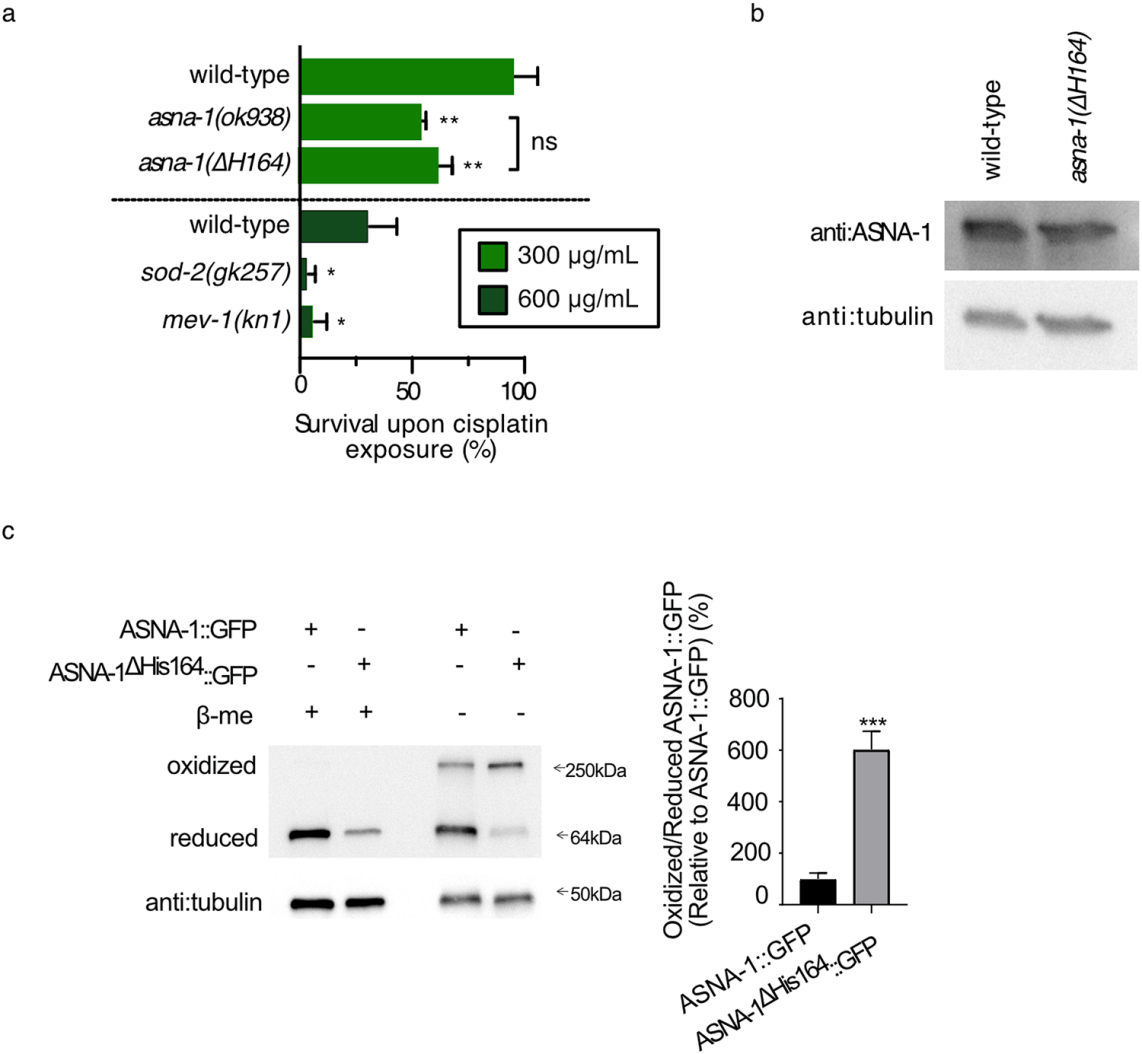
*asna-1* mutants with increased levels of ASNA-1^OX^ are cisplatin sensitive. (**a**) Mean survival ± SD of 1-day old adults after 24 h cisplatin (300 μg/mLor 600 μg/mL) exposure of worms with the indicated genotypes. Statistical significance was by the independent two-sample t-test (n ≥ 50). Experiments were performed in triplicate. (**b**) Western blot analysis after reduced SDS-PAGE of ASNA-1 levels in 1-day old adult wild-type and *asna-1*(*ΔHis164)* animals. The blot was probed with anti-ASNA-1 antibody and tubulin was used as a loading control. For full uncropped blot source images, see Supplementary Fig. S14. (**c**) Representative western blot after reducing and non-reducing SDS-PAGE to detect levels of oxidized and reduced ASNA-1^ΔHis164^::GFP. Control worms expressed the ASNA-1::GFP transgene. Blots were probed with anti-GFP antibody and tubulin was the loading control. For full uncropped blot source image, see Supplementary Fig. S14. Band intensity quantification of oxidized/reduced ASNA-1::GFP in different transgenic lines. Statistical significance was determined by the independent two-sample t-test. Experiments were performed in triplicate. Bars represent ± SD.

To obtain direct evidence for the separable functions of ASNA-1, we focused on the histidine 164 residue of ASNA-1 because of our previous work on the relevance of this amino acid for ASNA-1 function^7^. The *asna-1(ΔHis164)* mutation was generated by CRISPR-Cas9 technology, and the mutant was as sensitive to cisplatin as the *asna-1(ok938)* deletion mutant (Fig. 5a) while still producing normal levels of ASNA-1 protein (Fig. 5b). Furthermore, *asna-1(*Δ*His164)* mutants also displayed a strong TAP targeting defect (Supplementary Fig. S9). This conclusion was confirmed by the finding of decreased SEC-61β steady-state protein level (Supplementary Fig. S10), although as seen in *asna-1(ok938)* and *wrb-1(tm5938)* mutants the amount of glycosylated SEC-61β protein remained unchanged (Supplementary Fig. S3). We tested the redox balance of this mutant protein in worms expressing ASNA-1^ΔHis164^::GFP from a transgene. This transgene does not rescue the cisplatin sensitivity phenotype in *asna-1* null mutants but supports normal IIS activity^7^. Substantially more oxidized ASNA-1 was detected in this mutant compared to wild-type (Fig. 5c). Thus, an ASNA-1 mutant that displayed increased ASNA-1^OX^ was as sensitive to cisplatin as the deletion mutant. Consistently, *sod-2(gk257)* and *mev-1(kn1)* mutants, which displayed increased ASNA-1^OX^ levels (Fig. 1d,e), were sensitive to cisplatin (Fig. 5a) and *sod-2(RNAi)* animals displayed severe delocalization of the model TAP SEC-61β (Fig. 7d,e). Taken together, these lines of evidence support the notion that an oxidizing environment drives ASNA-1 into its oxidized state and results in a similar cisplatin sensitivity phenotype as a complete depletion of ASNA-1. We conclude that the ΔHis164 mutant in ASNA-1, which has inherently high ASNA-1^OX^ levels, is sensitive to cisplatin and has the TAP insertion defect but maintains normal insulin function. Hence the oxidized form is not required for cisplatin tolerance and TAP targeting. Driving ASNA-1 into the ASNA-1^OX^ state reduces insertion of a client TAP into the ER membrane without affecting insulin secretion.

### Cisplatin generates reactive oxygen species and promotes ASNA-1 oxidation

**Figure 6:**
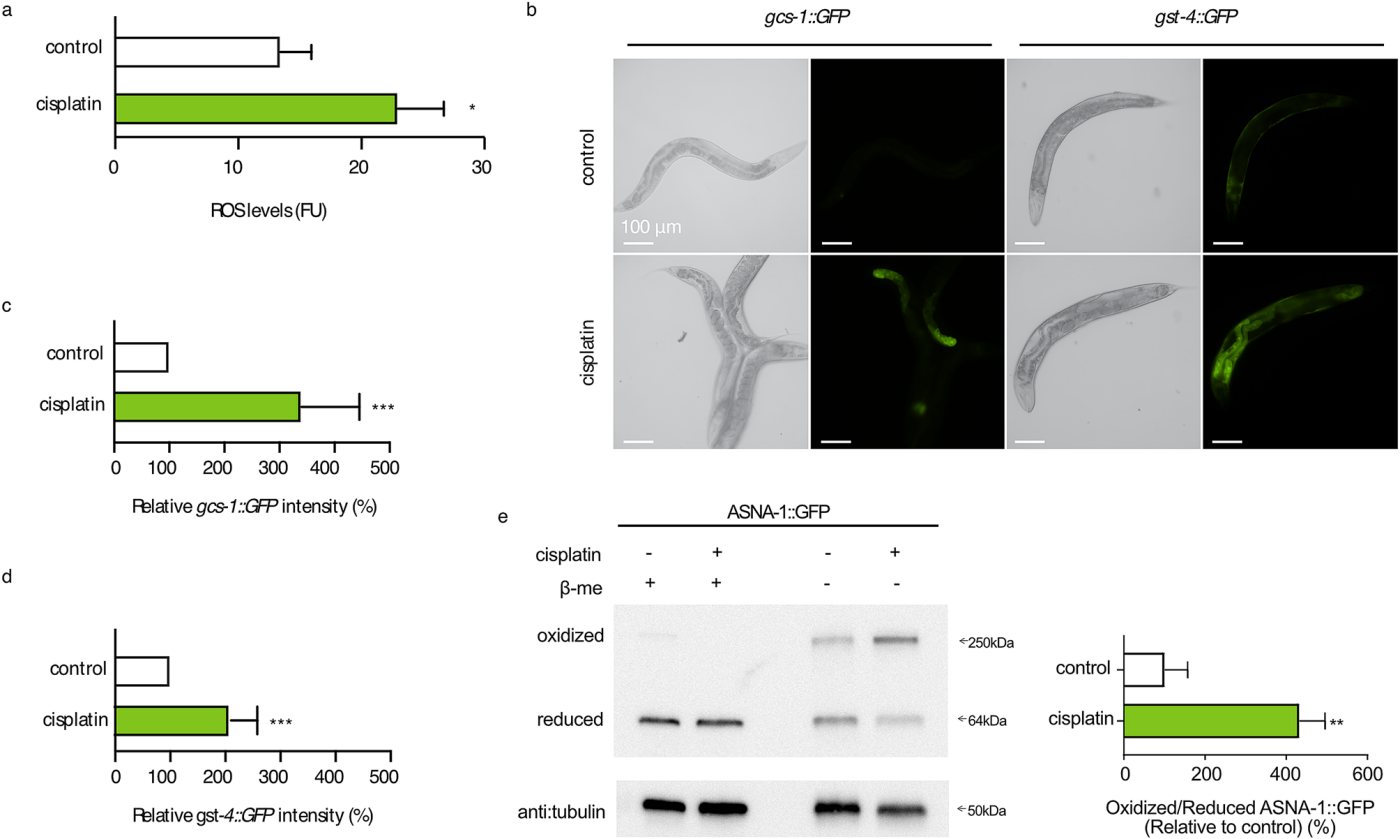
Cisplatin exposure elevates ROS levels and promotes ASNA-1 oxidation. (**a**) ROS production levels as estimated by the H_2_DCFDA assay on 1-day old adult wild-type animals (n ≥ 1000) was exposed to 600 μg/mL cisplatin for 1 h. Experiments were performed in triplicate. Statistical significance was determined by the independent two-sample t-test. Bars represent mean ± SD. (**b**) Increase of GFP intensity upon cisplatin exposure (300 µg/mL for 3 h) in *gcs-1::GFP* and *gst-4::GFP* transgenic strains. 1-day old adult animals were used in the analysis. (**c**) Quantification of relative GFP intensity for *gcs-1::GFP* (n ≥ 15) and (**d**) *gst-4::GFP* ( n ≥ 15) transgenic strains. Statistical significance was determined by the independent two-sample t-test. Bars represent mean ± SD. (**e**) Western blot analysis after reducing and non-reducing SDS-PAGE to detect oxidized and reduced ASNA-1::GFP in 1-day old adult animals exposed to cisplatin (300 µg/ml for 6 h). Blot was probed with anti-GFP antibody and tubulin was used as a loading control. For full uncropped blot source images including biological replicates used for quantification, see Supplementary Fig. S14. Band intensity quantification of oxidized/reduced ASNA-1::GFP exposed to cisplatin (300 µg/ml for 6 h). Statistical significance was determined by the independent two-sample t-test. Experiments were performed in triplicate. Bars represent ± SD.

Several groups have shown in mammalian cancer cell lines that cisplatin generates reactive oxygen species (ROS) and causes oxidative stress^32–35^. Having shown that ASNA-1 switches between functionally distinct redox states, we sought to examine if cisplatin affected the redox state of ASNA-1. We subjected worms to short cisplatin exposure regimens at 300 µg/mL or 600 µg/mL. Neither regimen decreased the viability of wild-type animals and thereby allowed meaningful cell biology analysis. These experimental conditions were indeed sufficient to induce ROS (Fig. 6a) and robustly activate the oxidative stress response genes *gcs-1* and *gst-4* (Fig. 6b–d). We next tested whether cisplatin exposure influenced the oxidation state of ASNA-1. ASNA-1::GFP-expressing worms were exposed to cisplatin at a concentration that significantly reduced the survival of *asna-1(ok938)* mutants without affecting wild-type animals (Fig. 5a). Under these conditions, the balance of ASNA-1::GFP shifted significantly towards the oxidized state (Fig. 6e). Thus, cisplatin-exposed worms displayed increased ROS levels and higher levels of oxidized ASNA-1 at the expense of the reduced form.

### Cisplatin selectively delocalizes an ASNA-1-dependent TAP from the ER

**Figure 7:**
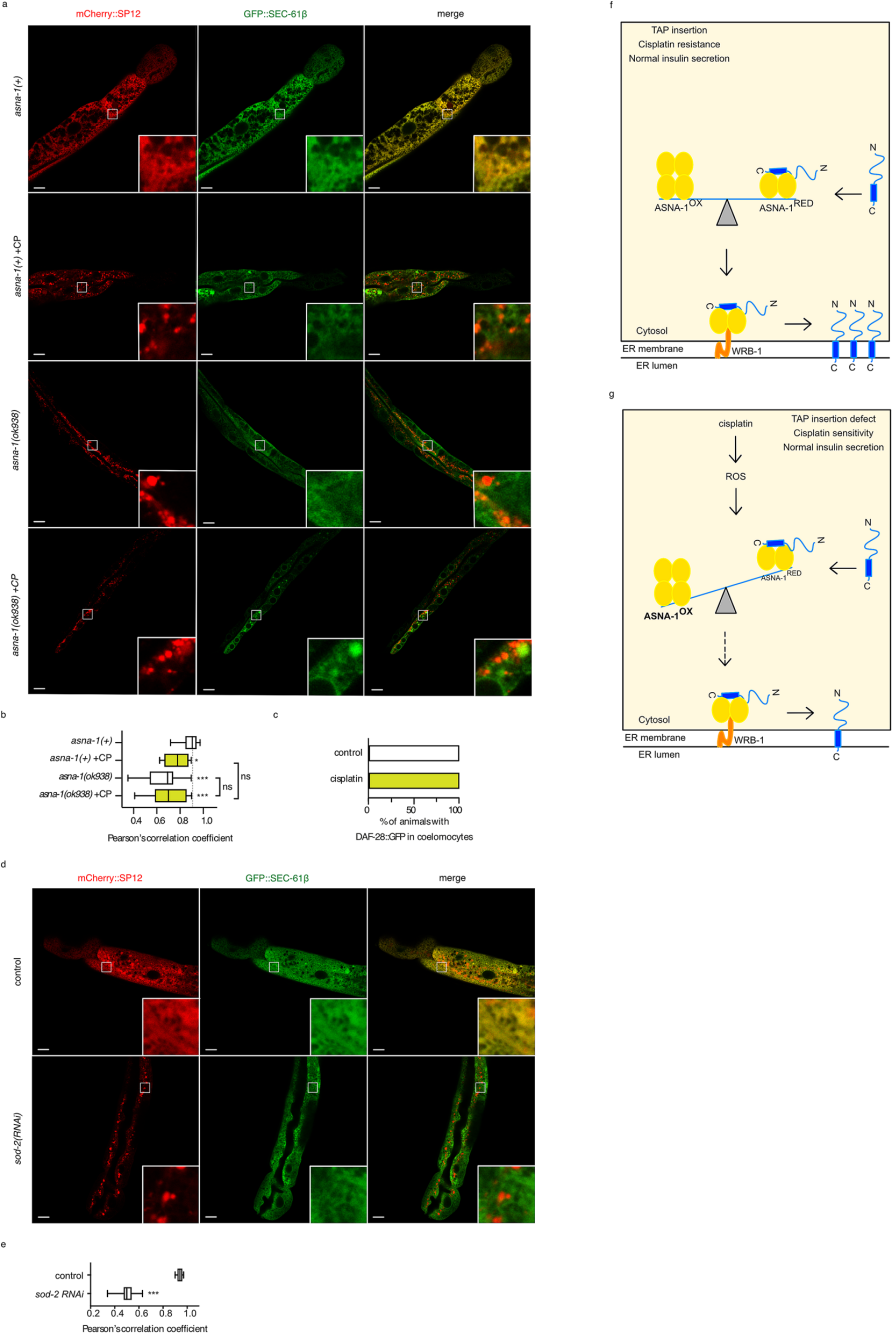
Cisplatin selectively delocalizes an ASNA-1-dependent TAP from the ER. (**a**) Representative confocal image of *asna-1(*+*)* or *asna-1(ok938)* 1-day old adult animals co-expressing GFP::SEC-61β with mCherry::SP12, without or with (+CP) cisplatin treatment (300 μg/mL for 6 h). Scale: 10 μm and 60 μm for magnification. Scale: 10 μm and 60 μm for magnification. (**b**) Pearson correlation analysis of GFP::SEC-61β and mCherry::SP12 co-localization. Box plot represent the average Pearson correlation coefficient (R) of the indicated strains without or with (+ CP) cisplatin treatment (300 μg/mL for 6 h). Statistical significance was determined by one-way ANOVA followed by Bonferroni post-hoc correction (n ≥ 10). (**c**) Bars represent percentage of 1-day old animals with secreted DAF-28::GFP in coelomocytes (n ≥ 20) exposed to cisplatin (300 µg/ml for 6 h). (**d**) Representative confocal images of empty vector or *sod-2(RNAi)* exposed 1-day old adult animals co-expressing GFP::SEC-61β with mCherry::SP12. Scale: 10 μm and 60 μm for magnification. (**e**) Pearson correlation analysis of GFP::SEC-61β and mCherry::SP12 co-localization. Statistical significance was determined by the independent two-sample t-test (n ≥ 10). (**f**–**g**) Model: under normal physiological conditions, ASNA-1 is present in both forms: reduced (ASNA-1^RED^) and oxidized (ASNA-1^OX^). ASNA-1^RED^ participates in the insertion of TAP into the ER membrane, while ASNA-1^OX^ assures proper insulin secretion (**f**). When the cells are exposed to cisplatin, elevated ROS levels perturb the ASNA-1 redox balance. ASNA-1 shifts towards the ASNA-1^OX^ state at the expense of ASNA-1^RED^, which impairs TAP targeting to the ER without affecting insulin secretion. Because less ASNA-1^RED^ is available, ER homeostasis is affected and cells are more sensitive to cisplatin (**g**).

Mindful of the role of the reduced form of ASNA-1 in TAP targeting, we next determined whether cisplatin treatment and TAP targeting were directly related. To this end, we tested whether the increased levels of oxidized ASNA-1 observed in cisplatin-treated worms resulted in a biologically meaningful outcome. Exposure of *asna-1(*+*)* animals expressing GFP::SEC-61β to cisplatin significantly delocalized this TAP away from the ER membrane at levels similar to those seen in untreated *asna-1(ok938)* animals (Fig. 7a,b). Remarkably, cisplatin treatment had no effect on the ER localization of the two ASNA-1-independent TAPs CytB5.1 (*cytb-5.1*) and SERP-1.1 (*serp-1.1*) (Supplementary Fig. S5). Therefore, delocalization of GFP::SEC-61β in cisplatin-treated worms was not due to a more generalized effect on membrane integrity. These results were confirmed by the observation that there was a reduced level of glycosylated SEC-61β upon cisplatin treatment (Supplementary Fig. S3) although the steady-state SEC-61β protein level remained unchanged (Supplementary Fig. S10). We next tested the effect of cisplatin exposure on DAF-28/insulin::GFP secretion and observed no secretion defect (Fig. 7c), indicating that membrane events associated with insulin maturation, packaging into dense core vesicles, and release were unaffected by cisplatin. Therefore, the effects of cisplatin on GFP::SEC-61β localization were not the result of a general toxicity. Since cisplatin oxidizes ASNA-1 and diminishes its TAP targeting capacity, we next asked whether the defective targeting of GFP::SEC-61β in cisplatin-treated animals was solely dependent on ASNA-1. When comparing targeting of GFP::SEC61β in *asna-1(ok938)* mutants with and without cisplatin treatment, we found that GFP::SEC61β was delocalized to the same extent in both conditions (Fig. 7a,b). Since there was no additive effect, we conclude that the diminished targeting of GFP::SEC61β caused by cisplatin was dependent on ASNA-1. Collectively these lines of evidence support a model (Fig. 7f,g) where cisplatin treatment generates ROS, in turn causing a rapid oxidation of ASNA-1 and a concomitant TAP targeting defect without affecting insulin secretion.

## Discussion

Only 5–10% of cisplatin is found in the nuclei of exposed cells, the remainder being cytoplasmic and membrane associated^35^. Non-nuclear cisplatin has been shown to modify kinase signaling, Ca^2+^ pump activity, and membrane dynamics^5,35^. In light of our findings that cisplatin modifies ASNA-1 function, it will be instructive to study which—if any—of these processes are caused by changes in ASNA-1 oxidation levels and ASNA-1-dependent TAP targeting. Our study shows that cisplatin has an effect on the ER in worm intestinal cells, which was not due to a general effect on cellular membranes at the concentrations of cisplatin used, since only an ASNA-1-dependent TAP was delocalized while ASNA-1-independent TAPs were targeted normally to the ER. Further, cisplatin exposure had no effect on DAF-28/insulin secretion, a process that also requires membrane budding and fusion events at various cellular locations. The normal insulin secretion levels observed in cisplatin-treated animals suggest that Golgi and plasma membranes are largely competent.

We characterized the TAP-targeting activity of *C. elegans* ASNA-1 and found that it possesses robust TAP targeting activity shared with its binding partner, the ER-localized protein WRB-1. *C. elegans asna-1* has all the structural features required for TAP insertion^10,36,37^. However, since not all ASNA-1 homologs have TAP-targeting activity^38^, it was important to examine whether *C. elegans* ASNA-1 has this function. In addition, parallel pathways like EMC, HSP40/HSC70, and SND participate in TAP targeting^23,39–41^. It was possible that compensatory TAP targeting by these pathways would make it difficult to detect a targeting defect in *asna-1* single mutants. Consequently, it was important to establish the in vivo contribution of ASNA-1 to TAP targeting in intact animals. We assayed this activity in large intestinal cells, because ASNA-1 is normally expressed in these cells and they are amenable to quantitative confocal microscopy. Our studies show that the other pathways do not bypass the need for ASNA-1 activity, since SEC-61β, which binds to ASNA-1, was significantly delocalized in *asna-1* mutants. We observed a significantly decreased steady-state level of SEC-61β which suggested degradation of the protein when insertion into the ER is inhibited, consistent with results from other studies^23,24^. A similar reduction in steady state levels of SEC-61β was seen also for the 3xFlag-tagged SEC-61β construct (Supplementary Fig. S3), confirming the results with the GFP construct. However, this does not rule out the function of the other pathways in intestinal cells. Importantly, using this assay, we also found ASNA-1-independent targeting of two ER-localized TAPs, in agreement with findings in other systems^14,15,42^. The contribution of other pathways to the targeting of ASNA-1-dependent TAPs in worms is unclear. It is possible that if TAP targeting is studied in *C. elegans* tissues other than the intestine, the EMC, SND or the HSP40/HSC70 pathways may play a greater role in the process.

*C. elegans* WRB-1 was identified as the closest homolog of mammalian WRB, containing three predicted trans-membrane domains and the important coiled-coil domain. Evidence that WRB-1 is the receptor for the ASNA-1/TAP complex comes from the fact that it was ER-localized and was an ASNA-1-binding protein, as are its homologs in other species^30,43^. Moreover, ASNA-1 protein levels were lower in *wrb-1* mutants, a property also seen upon mammalian WRB knockdown^44,45^. Taken together, these three properties of WRB-1 satisfy the requirement to regard this protein as the ER-based ASNA-1 receptor. Crucially, TAP localization was defective in two *wrb-1* mutants to a level similar to that seen in *asna-1* mutants. Moreover, a significant decrease in the steady-state SEC-61β protein level in both mutants was observed suggesting SEC-61β protein degradation when it is not inserted in the ER membrane. The observation that TAP targeting was defective in *wrb-1* mutants allowed us to address the role of ASNA-1 and WRB-1 in the biogenesis of TAP proteins with respect to insulin/IIS signaling. *wrb-1* mutants, unlike *asna-1* mutants, were not defective in insulin/IIS signaling and secretion function^8^, even though mutants in both genes have comparable levels of defective GFP::SEC-61β targeting. Taking into consideration the documented non-TAP insertion functions of ASNA-1/TRC40/GET3^11,17,46–49^, we think that reduction of ASNA-1 abundance in *wrb-1* mutants likely does not contribute to the *wrb-1* mutant phenotypes. Rather the non-overlapping *asna-1* and *wrb-1* mutants phenotypes provides further evidence for separation of functions of ASNA-1. These non-overlapping phenotypes are body size, extent of germline development and larval arrest in m^-^z^-^ genetic background. Additionally, Powis et al.^17^ showed that in the WRB homolog Get1 mutants, the wild type Get3 protein retains a robust holdase activity despite lowered protein levels. This demonstrates that the lowered levels of Get3 or ASNA-1 does not represent a mutant phenotype for non-TAP targeting roles. We cannot rule out the possibility that the reduced ASNA-1 levels might have an impact on the *wrb-1(-)* phenotypes. In order to test that hypothesis the analysis of double mutant (*asna-1;wrb-1*) should be carried out. Although, taking into consideration the essential role of both genes for animal survival, this approach will be challenging.

We concluded that ASNA-1-dependent TAP targeting has little role in ASNA-1-dependent DAF-28/insulin secretion. Moreover, this also meant that insulin/IIS signaling pathway defects do not contribute to the cisplatin hypersensitivity phenotype of *asna-1(-)* animals. This notion was consistent with our previous finding that *daf-2/insulin* receptor mutants, which are defective in IIS, are resistant to cisplatin^7^ and with our finding in this work that *unc-31/CAPS* and *tom-1/tomosyn* mutants have no effect on TAP targeting. Direct evidence that the cisplatin and insulin functions of ASNA-1 are separable emerged from the analysis of the *asna-1(ΔHis164*) mutant, which had the striking phenotype of completely separating the cisplatin and insulin secretion functions. *asna-1(ΔHis164*) animals are “null” for the cisplatin phenotype and “wild-type” for the growth and insulin secretion function of ASNA-1^7^. In contradiction, we previously observed that in lox/cre mediated knockdown Asna1^β−/−^ β-cells mis-localizes Stx5 and also leads to perturbance of retrograde transport and diminished insulin biogenesis which in turn leads to the development of diabetes^9^. We do believe that the differences between our studies might be attributed to the levels of ASNA-1 knock down in the two model systems. While *C. elegans* provides us with a model organism where we can study the relationship between the insulin secretion and TAP insertion in complete absence of ASNA-1, the Asna1^β−/−^ β-cells used in Norlin et al., 2016 had only diminished ASNA1 levels and about 40% of the protein was still present.

ASNA-1 is a member of a group of *C. elegans* proteins, along with PRDX-2, IRE-1, and GLB-12, whose activity changes with oxidation^50–52^. In vivo redox changes are dynamic in *C. elegans* with respect to both tissue type and age^53–55^. Oxidized proteins are likely to be abundant in the proteome, since quantitative redox proteomics has identified many proteins with redox-sensitive cysteines in H_2_O_2_-treated animals^56^ and in day 2 adults^57^. *C. elegans* ASNA-1 exists in both reduced and oxidized forms. This finding is consistent with the properties of the yeast homologue GET3 which exists as both a reduced, ATPase-dependent TAP targeting state and an oxidized ATPase-independent general chaperone/holdase state^11^. Moreover, the shift in the balance was strictly dependent on the presence of two conserved cysteines, which in GET3 assure the transition between open and closed state of the dimer^10^. A variant of GET3 in which those cysteines are mutated fail to dimerize and therefore shows no ATPase activity and severe tail-anchored binding defect^10,11^. Another redox sensitive protein playing a role in cisplatin resistance is the *C. elegans* TRXR-1, which contains a selenocysteine^58^. Its mammalian homolog can be targeted by cisplatin to form cytotoxic SecTRAPS^59^. Work in *C. elegans* shows that TRXR-1 confers systemic sensitivity to cisplatin via the selenocysteine residue in larval stage animals displaying mitotic proliferation^60^.

The ASNA-1^RED^/ASNA-1^OX^ balance was biologically relevant, since it was sensitive to changes in animal physiology. Worm mutants with high ROS levels or cultivation conditions that increase ROS shifted the balance towards more oxidized ASNA-1, while exposure to an antioxidant shifted the balance in the opposite direction. Interconversion between the two redox states was observed and levels of ASNA-1^OX^ increased with a corresponding decrease in levels of ASNA-1^RED^ and vice versa. Interestingly, ASNA-1^ΔHis164^ worms displayed high ASNA-1^OX^ levels and were cisplatin sensitive. Analysis of this mutation reveals the separation of functions since transgenic expression of ASNA-1^ΔHis164^ does not rescue cisplatin hypersensitivity of the null mutant but maintains largely intact insulin/IGF signaling^7^. Moreover, *sod-2* mutants, which were cisplatin sensitive and showed increased ASNA-1^OX^ levels, also exhibited a SEC-61β delocalization defect, further supporting the notion that a shift toward ASNA-1^OX^ at the expense of ASNA-1^RED^ form will lead to cisplatin sensitivity and TAP targeting defects. This implies that, likely the inadequate levels of ASNA-1^RED^ would lead to cisplatin sensitivity. Further studies on worm mutants producing only functional ASNA-1^RED^ would confirm this.

Worms expressing ASNA-1 where the two conserved cysteines are mutated (ASNA-1^C285S;C288S^) is not oxidized. However the GET3^C285S;C288S^ form of the protein fails to dimerize for TAP targeting function and be oxidized for its general chaperone function^10,11^. Therefore, this mutation affects all protein functions and makes it impossible to study the only ASNA-1^RED^ in the absence of ASNA-1^OX^. However, while we cannot rule out the role of ASNA-1^OX^ in cisplatin detoxification, our work shows that ASNA-1^OX^ is not sufficient for this function.

Most cells in human solid tumors are in a post-mitotic state^1^, as is the case in adult worms. Given the range of effects exerted by cisplatin on cellular function, the killing effect can not only be attributed to induced DNA damage but is also likely to be partially due to effects on the ER. We have shown that increased levels of oxidized ASNA-1 beyond a certain threshold will sensitize cells to cisplatin. We propose that drugs that increase oxidized ASNA-1 at the expense of the reduced form will enhance cisplatin cytotoxicity. This work also reveals for the first time that cisplatin impacts TAP targeting. The TAP pathway has been studied in detail and the roles of several other proteins besides ASNA1/TRC40 have been delineated. It is likely that drugs affecting other components of the TAP targeting pathway would also increase cisplatin sensitivity. This analysis of ASNA-1 demonstrates that cisplatin perturbs ER function, which might also explain other effects of cisplatin on signaling pathways, Ca^2+^ homeostasis, and membrane properties. We previously showed that, while cisplatin does not induce ER stress, combination use with an ER stress inducer sensitizes resistant worms to cisplatin^26^. This is consistent with the idea that cisplatin treatment sensitizes the ER to a metastable state due to TAP targeting defects and if ER function is further compromised, then the cells will become hypersensitive to cisplatin. Drugs that increase ASNA-1 oxidation, target other components of the TAP biogenesis pathway, or induce mild effects on ER function may enhance cisplatin sensitivity and address the problem of cisplatin resistance.

## Material and methods

### *Caenorhabditis elegans* genetics and maintenance

The Bristol strain (*N2*) was the wild-type. N2 and TJ356, carrying *zIs356(daf-16::gfp)*, are described in WormBase (http://www.wormbase.org). *wrb-1(tm5938 & tm5532)* were obtained from the Mitani lab and NBRP, Tokyo (http://www.shigen.nig.ac.jp/c.elegans/index.jsp). *wrb-1(tm5938)* was outcrossed 6 times and maintained in trans to the *nT1(qIs51)* balancer. *svIs135 [Pvha-6::gfp::sec-61.B(Y38F2AR.9)* + *Pvha-6::mCherry::SP12]* was obtained by genomic integration of an extrachromosomal array generated by microinjection of pVB639OB and pVB641OB at 50 ng/μl each. *svIs135* bearing worms were outcrossed 6 times before use. *svIs143(Pnhx-2::mCherry::lgg-1)* was generated by genomic integration of *vkEx1093*^61^ and outcrossed 3 times before use. *svEx917* (*Pvha-6::gfp::cytb-5.1; Pvha-6::mCherry::SP12*) was obtained by injecting pVB640OB and pVB641OB at 50 ng/μl each into N2. RNAi was performed as described^62^. The transgenic worms carrying *svIs56* (*asna-1::gfp)* and *svIs69(daf-28::gfp)* have been described^8^. The integrated transgene *rawIs13* expressing *asna-1*^*C285S;C288S*^*::GFP* was obtained by gamma ray irradiation of worms bearing the *svEx756* extrachromosomal transgene^7^. The single copy *asna-1::gfp (knuSi184)* contained 1.4 kb upstream promoter sequence driving genomic *asna-1* coding region fused to GFP just before the stop codon followed by the *tbb-2* 3′ UTR. This construct was inserted on chromosome II at the *ttTi5605* locus using MosSCI technology^63^ by Knudra Transgenics. *sod-2(gk257*), *mev-1(kn-1*), *hsp-4::GFP (zcIs4*), *unc-119(ed3);oxTi880, gcs-1::GFP* (*ldIs3*) and *gst-4::GFP (dvIs19)* were obtained from Caenorhabditis Genetics Center (CGC) (http://www.cgc.umn.edu). The GFP::TAP plasmids were co-injected with pVB641OB (each at 50 ng/μL) to generate strains to assay TAP localization to the ER. *svIs135* expressed GFP::SEC-61β, *svEx917* expressed GFP::CYTB-5.1, *rawEx14* expressed GFP::SERP-1.1, *rawEx21* expressed GFP::SEC-61β^ΔTMD^. *rawEx64* expressed *Pvha-6*-*3xFlag::SEC-61β*::*opsin* as an extrachromosomal array and was outcrossed 2 times before use. *rawIs19* (*Pvha-6*-*3xFlag::SEC-61β*::*opsin*) was generated by genomic integration of *rawEx64* and outcrossed 4 times before use. *rawIs19* likely inserted near the *asna-1* locus and therefore the *rawEx64* transgene was used in combination with *asna-1* mutants. The *asna-1(ΔHis164)* mutation *syb1544,* was obtained by deletion of histidine 164 using CRISPR-CAS9 technology by Sunybiotech and was maintained over *hT2(qIs48)*. The ASNA-1^ΔHis164^::GFP expressing transgene was *svEx591*^7^. See Supplementary Table S2 for more details.

### TAP-targeting analysis

Live 1-day old adult animals were sedated in 2 mM Levamisole/M9 and mounted onto 2% agarose pads. The int8 and int9 cells in the posterior intestine were imaged. The fluorescence signals were analyzed at 488 nm and 555 nm by the LSM700 Confocal Laser Scanning Microscope (Carl Zeiss) with LD C-Apochromat 40×/1.1 W Corr. objective. The gain setting was adjusted to remove signal from intestinal autofluorescence in wild-type worms that were analyzed with the same parameters as transgenic worms, to ensure that no autofluorescence signal was detected (Supplementary Fig. S15). Pixel size and pinhole settings were identical for all images. Image processing of Z-stacks was performed with the ZEN Lite program. (Zeiss) Correlation quantification was done using Volocity software (version 6.3, PerkinElmer, Coventry, UK, http://www.perkinelmer.com). Correlation quantification was done using Automatic Thresholding^64^ method to set thresholds objectively. For DTT exposure, washed worms were placed on seeded NGM plates containing 10 mM DTT for 4 h before imaging. Plates from the same batch were tested for P-hsp-4::GFP induction in *zcIs4 (Phsp-4::gfp)* worms under the same conditions to ensure that UPR was properly induced. Starvation was induced by placing extensively washed worms for four hours on unseeded NGM plates before imaging.

### Insulin assays

Larval arrest phenotypes were scored in the F1 generation from *wrb-1* dsRNA-injected mothers grown at 20 °C. Worms harboring integrated *daf-16::gfp(zIs356)* and *daf-28::gfp(svIs69)* arrays were grown at 20 °C and imaged using a Nikon microscope, equipped with Hammamatsu Orca flash4.0 camera. *daf-16::gfp* animals were analyzed within 10 min after mounting to avoid artifacts due to stress. DAF-28::GFP uptake by coelomocytes was scored in adult worms as described^8^.

### Glycosylation analysis of tagged SEC61β

Young adult worms expressing *3xFlag::SEC-61β*::*opsin* from the *rawIs19* or *rawEx64* transgenes (Fig. S3) were homogenized and taken for protein concentration determination. The lysate was split in two and one half treated with Endoglycosidase H (V4871, Promega) as described by the supplier. All samples were solubilized in Laemmli buffer, separated by SDS-PAGE followed by western transfer and detection by immunoblotting with anti-Flag [F1804] (Sigma) antibody.

For the sterile mutants expressing either transgene, homozygotes were hand-picked and solubilized in Laemmli buffer and the lysates were subjected to SDS-PAGE and western blot analysis as above.

### Western blot analysis

#### Reducing SDS-PAGE

Synchronized young adult worms were homogenized and protein concentration determined using the BCA assay (Thermo Scientific). Samples were boiled for 10 min in reducing loading buffer (SDS/β-mercaptoethanol). Proteins were separated by SDS-PAGE and blotted onto PVDF membranes *Non-reducing SDS-PAGE:* Lysates were boiled for 10 min in non-reducing (without β-mercaptoethanol) loading buffer and cooled to room temperature for 10 min. To protect free cysteine thiols from post-lysis oxidation, iodoacetamide was added to the samples at a final concentration of 25 mM followed by a 30 min incubation in darkness at room temperature. Proteins were separated by SDS-PAGE and blotted onto nitrocellulose membranes. *Antibodies:* anti-ASNA-1 antibody^8^, anti-GFP antibody [3H9] (Chromotek) and anti-Flag antibody [F1804] (Sigma) were used. To assess equal loading, membranes were stripped and probed with anti-alpha tubulin (T5168, Sigma). Band quantification was performed using ImageJ software^65^.

### Subcellular fractionation

Young adult animals grown at 20 °C were harvested, washed and lysed in extraction buffer (50 mM Tris, pH7.2, 250 mM sucrose, 2 mM EDTA). Supernatants were centrifuged for 60 min at 100,000×*g* at 4 °C. The supernatant fraction was concentrated using Vivaspin Concentrators (Sigma). The pellet fraction was resuspended in 1× Laemmli buffer (Biorad). Proteins in both fractions were separated by SDS-PAGE and blotted onto PVDF membranes. Proteins were detected using anti-GFP antibody [3H9] (Chromotek) or anti-alpha tubulin [T5168] (Sigma).

### Pro-oxidant, anti-oxidant treatment and cisplatin treatment

#### H_2_O_2_

Harvested adult worms were washed and incubated with 5 mM H_2_O_2_ for 30 min, followed by lysis and western blot analysis. For recovery from H_2_O_2_ exposure, washed worms were put onto seeded NGM plates for the indicated times. *Epigallocatechin gallate (EGCG)*: EGCG (Sigma) containing plates were prepared by spreading 200 µL of 400 µM EGCG dissolved in water on unseeded NGM plates to obtain a final concentration of 5.7 µM. Spots of concentrated OP50 were applied for food after the plates had dried. L3 larvae were transferred onto NGM + EGCG plates for 24 h and transferred onto fresh EGCG plates for another 24 h. Worms were washed 3 times with M9 followed by lysate preparation or exposed to cisplatin and tested for survival. *Cisplatin treatment:* Cisplatin plates were prepared using MYOB media with 2% agar in which the drug was added at a final concentration of 300 μg/mL. Cisplatin solution (1 mg/mL, Accord Healthcare AB) was added to autoclaved medium after cooling to 52 °C. Young adults (24 h post L4 stage) were collected, washed and incubated on cisplatin plates for 3 or 6 h. Worms were harvested and processed for lysate preparation.

### Cisplatin sensitivity assay

Cisplatin plates were prepared as described above. L4 larvae were isolated and grown for 24 h before exposure to cisplatin. After 24 h cisplatin exposure, death was determined by absence of touch-provoked movement when stimulated by harsh touch using a platinum wire.

### RNA isolation and quantitative PCR

Total RNA was extracted using Aurum Total RNA Mini Kit (BioRad). cDNA was synthesized using iScript cDNA Synthesis Kit (BioRad). qPCR was performed on a CFX Connect machine (BioRad) instrument using KAPA SYBR FAST qPCR Kit (KapaBiosystems) with the comparative Ct method and normalization to the housekeeping gene *F44B9.5*. All samples were tested in triplicates.

### Immunoprecipitation

Mixed stage worms expressing ASNA-1::GFP were harvested and lysed in lysis buffer (10 mM Tris/Cl, 150 mM NaCl, 0.5 mM EDTA, 0,5% NP-40). Lysates were cleared and protein concentrations determined as before. Lysate containing 5000 μg total protein was added to GFP-Trap MA magnetic beads (Chromotek) and tumbled end-over-end for 1 h at 4 °C. Beads were magnetically separated, washed in wash buffer (0.01% Tween-20 in 50 mM triethylammonium bicarbonate (TEAB)), followed by three washes in 50 mM TEAB. Elution of protein from the beads was performed by adding 0.2 M glycine (pH 2.5), followed by magnetic separation. 1 M Tris base (pH10.4) was added for neutralization. The GFP expressing strain (*unc-119(ed3);oxTi880*) was used as a negative control for GFP-interacting partners. Experiment was performed three times for identification of WRB-1 as an interacting partner.

### ROS estimation assays

Reactive oxygen species (ROS) levels were quantified using 2,7-dichlorodihydrofluorescein diacetate (H_2_DCFDA, Thermo Scientific). *For cisplatin treatment*: Approximately 1000 young adult worms were incubated on OP50 containing MYOB/cisplatin plates for 3 h. Worms were harvested, and cleaned from bacteria, concentrated in approximately 100 μL M9 and transferred to an assay well of a 96 well plate. 100 μL of 100 μM of H_2_DCFDA was added to achieve a final concentration of 50 μM. Non-treated animals were similarly processed. Fluorescence was read at the time of adding the dye and one hour after dye addition, using a fluorimeter {485 excitations, 520 emissions}. Initial readings were subtracted from the final readings and fluorescence per 1000 worms was calculated.

### Statistical analysis

Statistical analysis was performed with Prism 7 software (GraphPad software version 8.4.3 for Windows, La Jolla, CA, USA, http://www.graphpad.com). P-values indicated statistical significance (*p < 0.05, **p < 0.01, ***p < 0.001).

## Supplementary Information

Supplementary Information 1.

Supplementary Information 2.

## Data Availability

All data is available in the main text or the supplementary materials.
