# Supplementary material for: Alternative redox forms of ASNA-1 separate insulin signaling from tail-anchored protein targeting and cisplatin resistance in C. elegans
Source: Sci Rep. 2021 Apr 21;11:8678. doi: 10.1038/s41598-021-88085-y (PMC8060345; doi:10.1038/s41598-021-88085-y)
Supplement: Supplementary file 1 — Supplementary Information 1. [file 41598_2021_88085_MOESM1_ESM.pdf]

Alternative redox forms of ASNA-1 separate insulin signaling from tail-anchored protein targeting and cisplatin resistance in *C. elegans*

Dorota Raj[1, †], Ola Billing[2, †], Agnieszka Podraza-Farhanieh[1, †], Bashar Kraish[1], Oskar Hemmingsson[2], Gautam Kao[1,*] and Peter Naredi,[1,3,*]

[1]Department of Surgery, Institute of Clinical Sciences, Sahlgrenska Academy, University of Gothenburg, SE413 45 Gothenburg, Sweden
[2]Department of Surgical and Perioperative Sciences, Surgery, Umeå University, SE901 85 Umeå, Sweden
[3]Department of Surgery, Sahlgrenska University Hospital, SE413 45 Gothenburg, Sweden
†These authors contributed equally

***Corresponding author:** peter.naredi@gu.se; gautam.kao@gu.se

SUPPLEMENTARY FIGURES

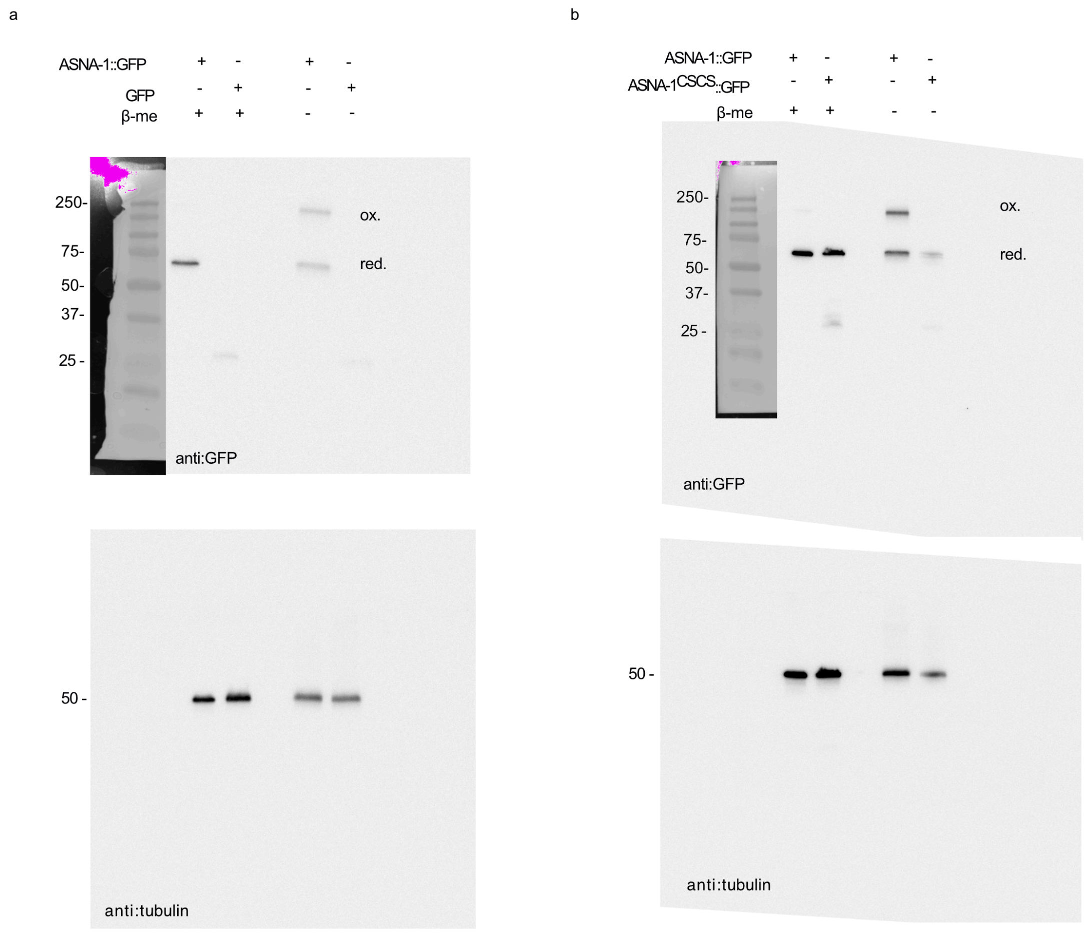

Figure S1. Full uncropped images of Western blots following reducing and non-reducing SDS-PAGE to detect (a) oxidized and reduced ASNA-1::GFP in 1-day old adult animals expressing multicopy ASNA-1::GFP and strain expressing only GFP *(unc-119(ed3);oxTi880)*; (b) in *rawIs13* transgenic worms expressing ASNA-1$^{C285S;C288S}$ ::GFP ; Blots were probed with anti-GFP antibody and tubulin was used as a loading control. Ox – oxidized ASNA-1::GFP, red. – reduced ASNA-1::GFP.

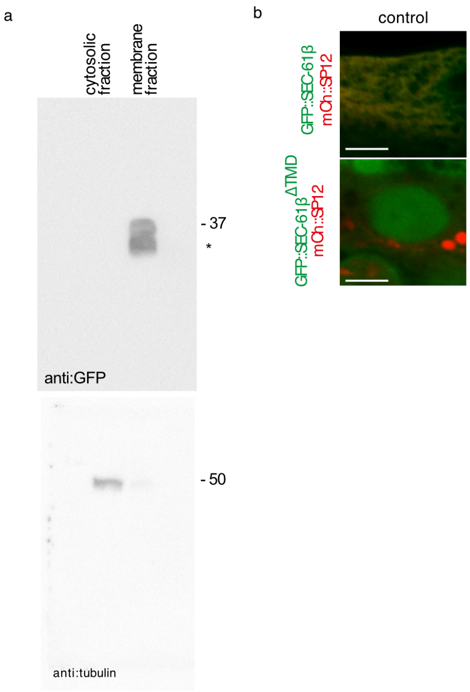

Figure S2. (a) Full uncropped images of Western blot following reducing SDS-PAGE to detect subcellular localization of GFP::SEC-61β. Blot was probed with anti-GFP antibody. Tubulin used as a loading control. Asterisk (*) indicates non-specific band. (b) Confocal imaging merge of 1-day old animals co-expressing GFP::SEC-61β or GFP::SEC-61$\beta^{\Delta TMD}$ (deleted for the trans membrane domain) with mCherry::SP12. Scale: 5 µm.

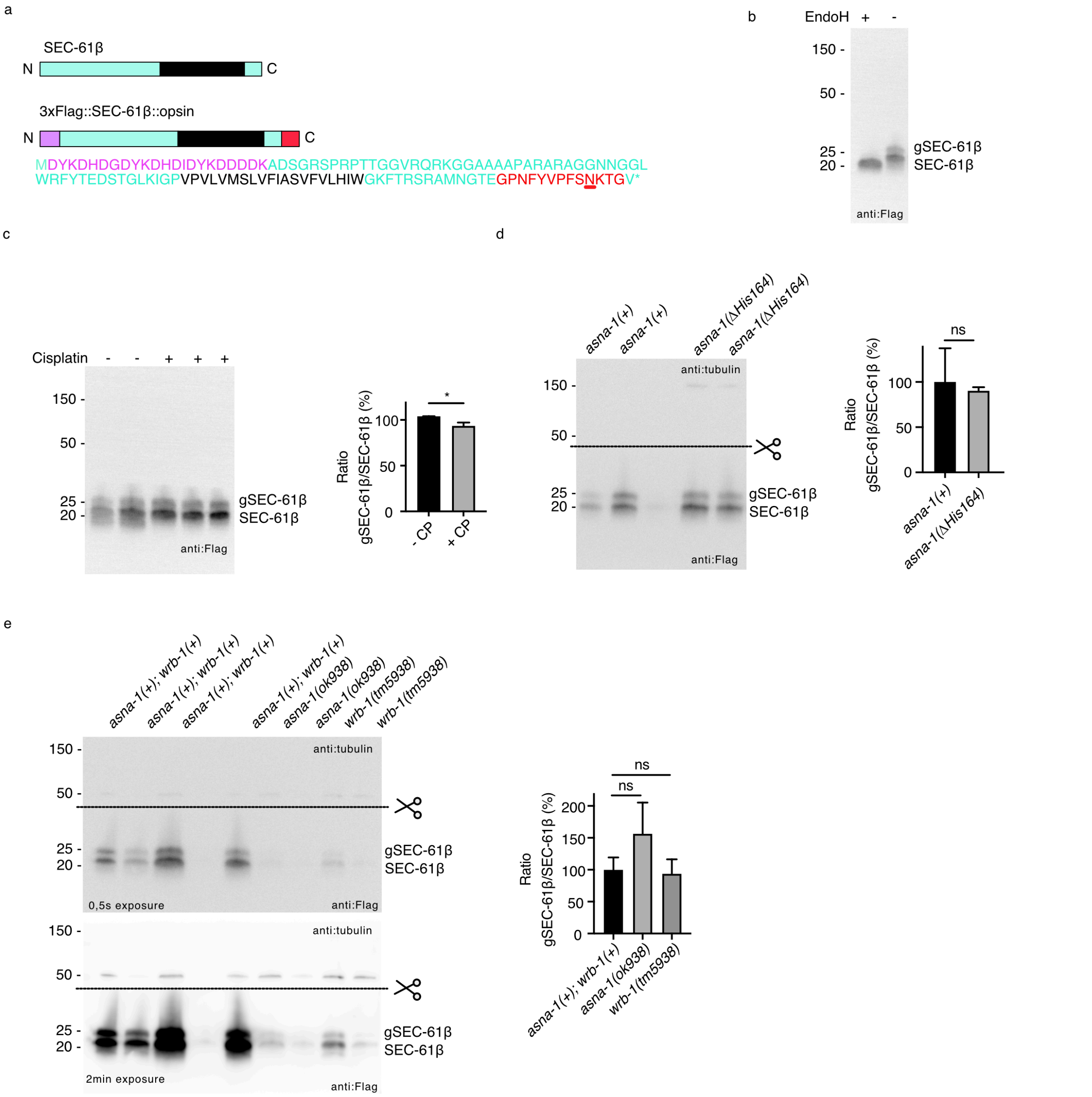

Figure S3. (a) Schematic representation of SEC-61β and 3xFlag::SEC-61β::opsin. In plasmid pGK213, 3xFlag::SEC-61β::opsin contains at its N-terminus a 3xFlag tag (purple box and lettering) and at its C-terminus a bovine opsin tag containing the reactive asparagine that is the acceptor for N-linked glycosylation (red box an lettering). Underlined Asparagine (N) marks glycosylation site. The transmembrane domain (TMD) is represented as a black box and lettering. (b) Glycosylated (gSEC61β) and non-glycosylated (SEC61β) proteins were resolved by SDS-PAGE, subjected to western blotting detected by anti-Flag antibody. The identity of glycosylated band identity was confirmed by endoglycosidase H (EndoH) treatment. (c-e) Full uncropped images of western blot following reducing SDS-PAGE to detect glycosylation of SEC-61β in strains carrying 3xFlag::SEC-61β::opsin (c) *rawIs19* non-exposed or exposed to

300 µg/mL cisplatin for 6h; (d) in *rawEx64* and *asna-1(ΔHis164);rawEx64*; (e) in *rawEx64, asna-1(ok938);rawEx64* and *wrb-1(tm5938);rawEx64*. Dotted line represents place of membrane cut in order to simultaneously probe with anti-Flag or anti-tubulin antibody. Statistical significance was determined by the independent two-sample t-test. Bars represent mean ± SD.

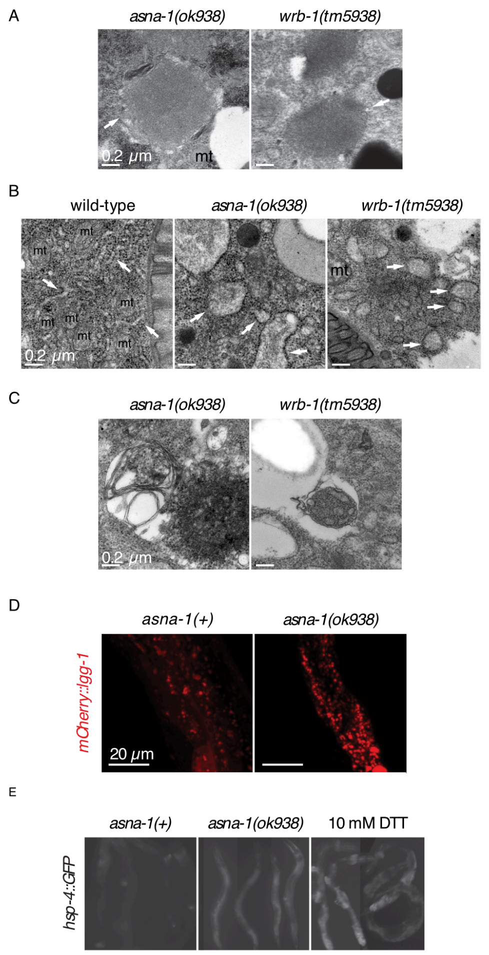

Figure S4. (a) Examples of cytosolic inclusion bodies (white arrows) found in the intestinal cells of *asna-1(ok938)* and *wrb-1(tm5938)* animals. The white arrows highlight examples of membrane whorls that in some cases flank the inclusion bodies in intestinal cells of *asna-1(ok938)* animals. (b) Dilated ER lumen in intestinal cells of *asna-1(ok938)* and *wrb-1(tm5938)* animals. White arrows indicate rough ER (RER) membranes. (c) Micrograph showing portions of the RER engulfed in autophagosomes (ERAs) in *asna-1(ok938)* and *wrb-1(tm5938)* animals. (d) Confocal images of representative adult animals expressing mCherry::lgg-1 from the *svIs143* transgene. mCherry::LGG-1 localization in *asna-1(+)* (left panel) is more diffuse and less punctate compared to that in *asna-1(ok938)* animals (right panel). (e) Expression from the Phsp-4::GFP reporter (*zcIs4*) imaged by fluorescence microscopy. The reporter induction in *asna-1(ok938)* mutants is comparable to the 10 mM DTT exposure for 4 h in wild-type worms. Animals undergoing the L3/L4 molt were excluded from the analysis, since molting caused a sharp increase in Phsp-4::GFP expression.

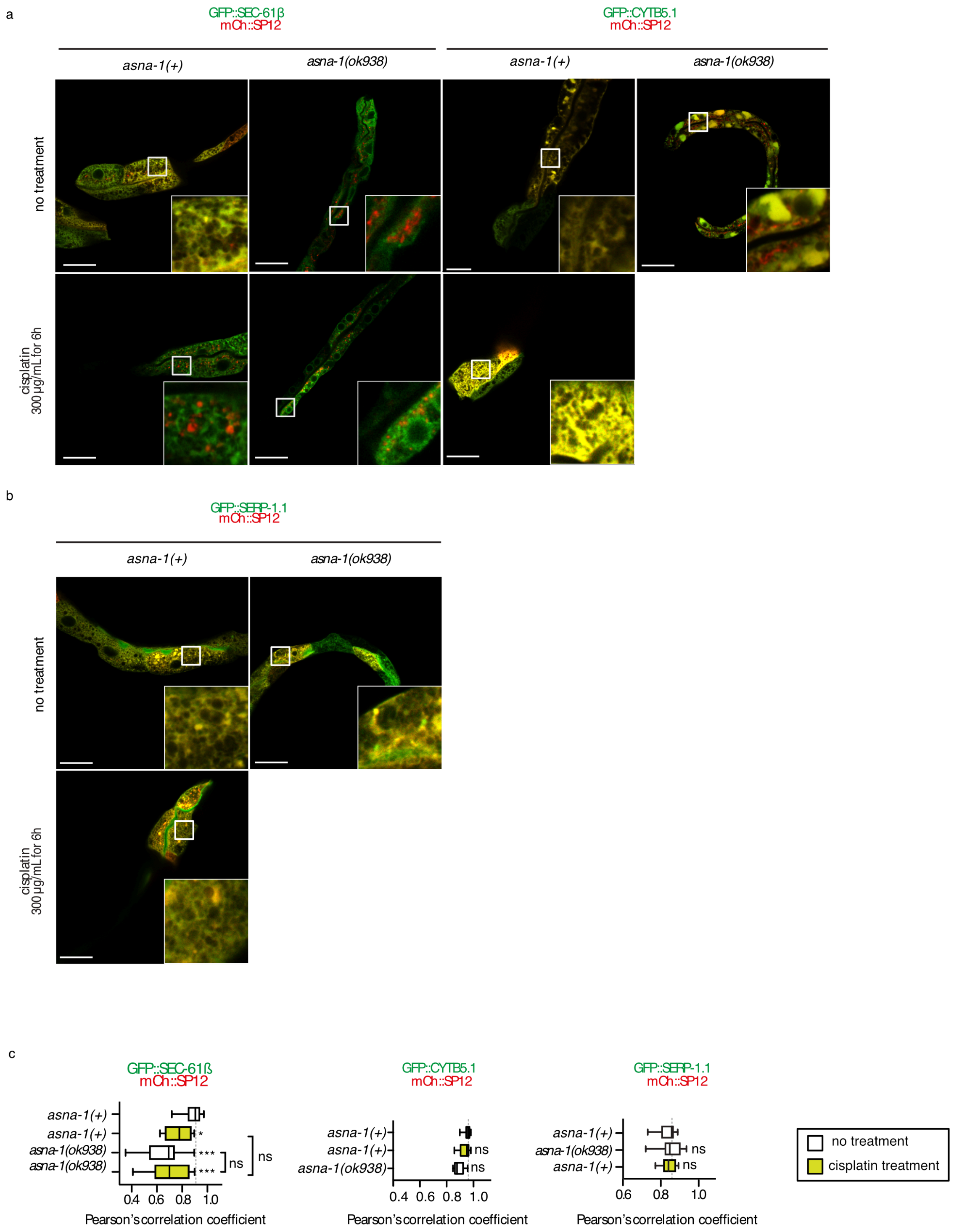

Figure S5. Representative confocal imaging merge of 1-day old *asna-1(+)* or *asna-1(ok938)* animals co-expressing (a) GFP::SEC-61β, GFP::CYTB5.1, or (b) GFP::SERP-1.1 with mCherry::SP12, with or without cisplatin treatment (300 µg/mL for 6 h). Scale: 5 µm and 20 µm for magnification.

(c) Pearson correlation analysis of colocalization of GFP::SEC-61β, GFP::CYTB5.1, or GFP::SERP-1.1 with mCherry::SP12. Box plot represent the average Pearson correlation coefficient (R) of the indicated strains without or with cisplatin treatment (300 μg/mL for 6 h). Statistical significance was determined by the independent two-sample t-test ($n \geq 10$) or one-way ANOVA followed by Bonferroni post-hoc correction ($n \geq 10$).

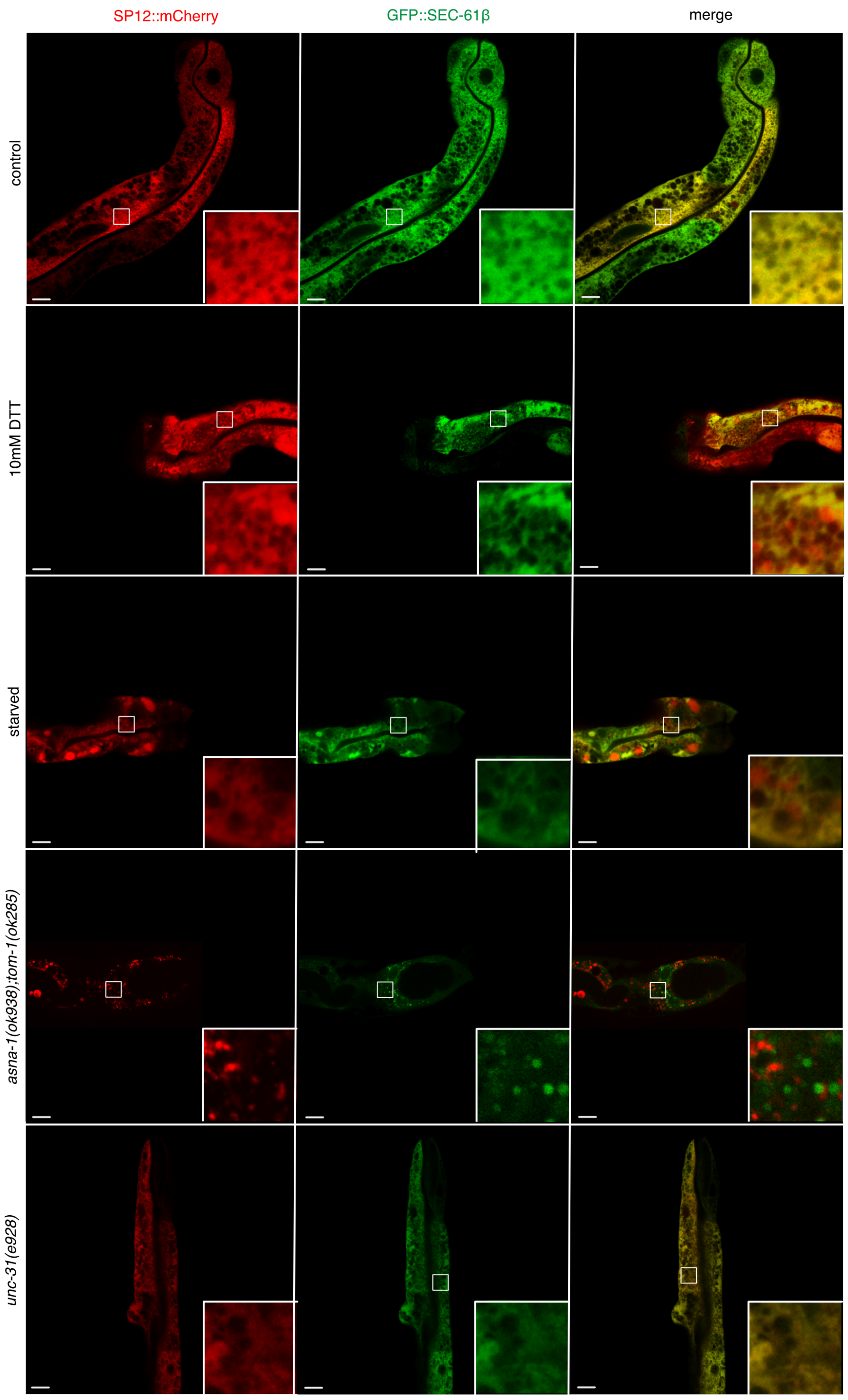

Figure S6. Representative confocal image of 1-day old animals co-expressing GFP::SEC-61β and mCherry::SP12 with the indicated treatment and genetic backgrounds. Scale: 10 μm and 60 μm for magnification.

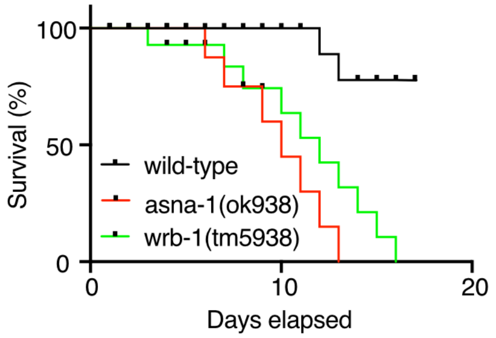

Figure S7. Life span analysis of wild-type (n=20), *asna-1(ok938)* (n=20) and *wrb-1(tm5938)* (n=20) animals.

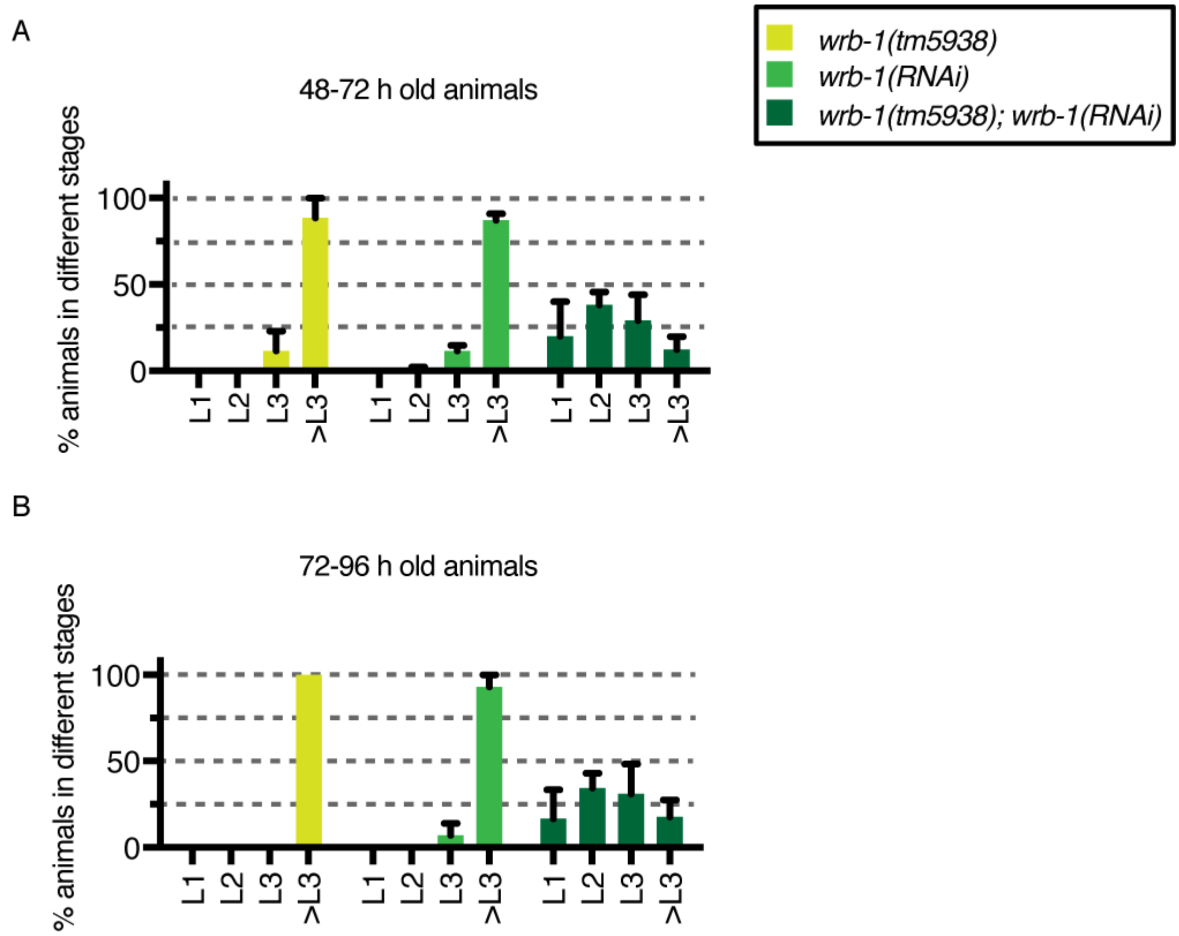

Figure S8. *wrb-1(tm5938)* animals were injected with *wrb-1* dsRNA into the gonads and allowed to lay eggs for 24 h before being removed. Animals were scored after 48 h (a) and 72 h (b) by counting the number of animals in each stage. Bars represent mean ± SD.

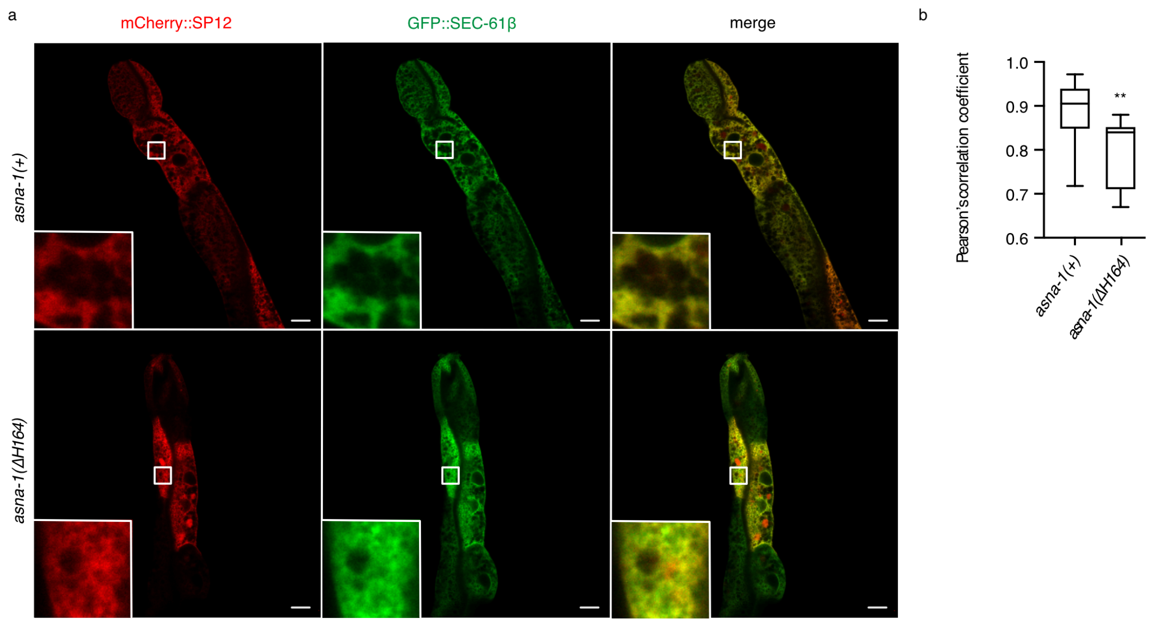

Figure S9. (a) Representative confocal image of *asna-1(+)*, and *asna-1(ΔHis164)* 1-day old adult animals co-expressing GFP::SEC-61β with mCherry::SP12 in intestinal int8 and int9 cells. Scale: 10 µm and 60 µm for magnification. (b) Pearson's correlation analysis of GFP::SEC-61β and mCherry::SP12 co-localization in different strains. Box plot represents the average Pearson correlation coefficient (R) of the indicated strains. Statistical significance was determined by Mann-Whitney test ($n \geq 10$ in all cases).

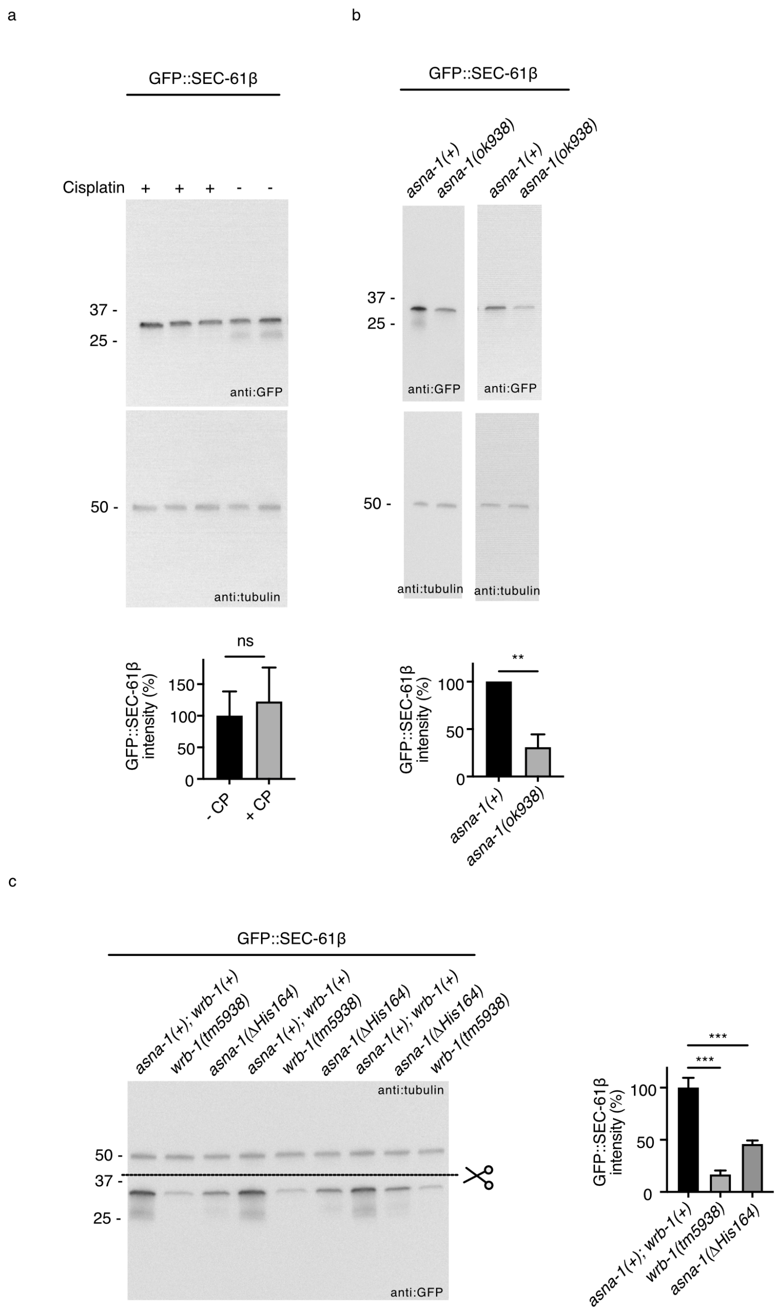

Figure S10 Full uncropped images of western blot following reducing SDS-PAGE to detect steady-state levels of GFP::SEC-61β expressed from the *svIs135* transgene (a) in *asna-1(+)* animals unexposed or exposed to 300 µg/mL cisplatin for 6h; (b) in *asna-1(+)* and *asna-1(ok938)* mutants; (c) in *asna-1(+)*;*wrb-1(+)*, *wrb-1(tm5938)* and *asna-1(ΔHis164)* mutants. Blots were probed with anti-GFP antibody. Tubulin used as a loading control. Statistical significance was determined by the independent two-sample t-test (a and b) and paired sample t-test (c). Bars represent mean ± SD. Dotted line in (c) represents place of membrane cut in order to simultaneously probe with anti-GFP or anti-tubulin antibody.

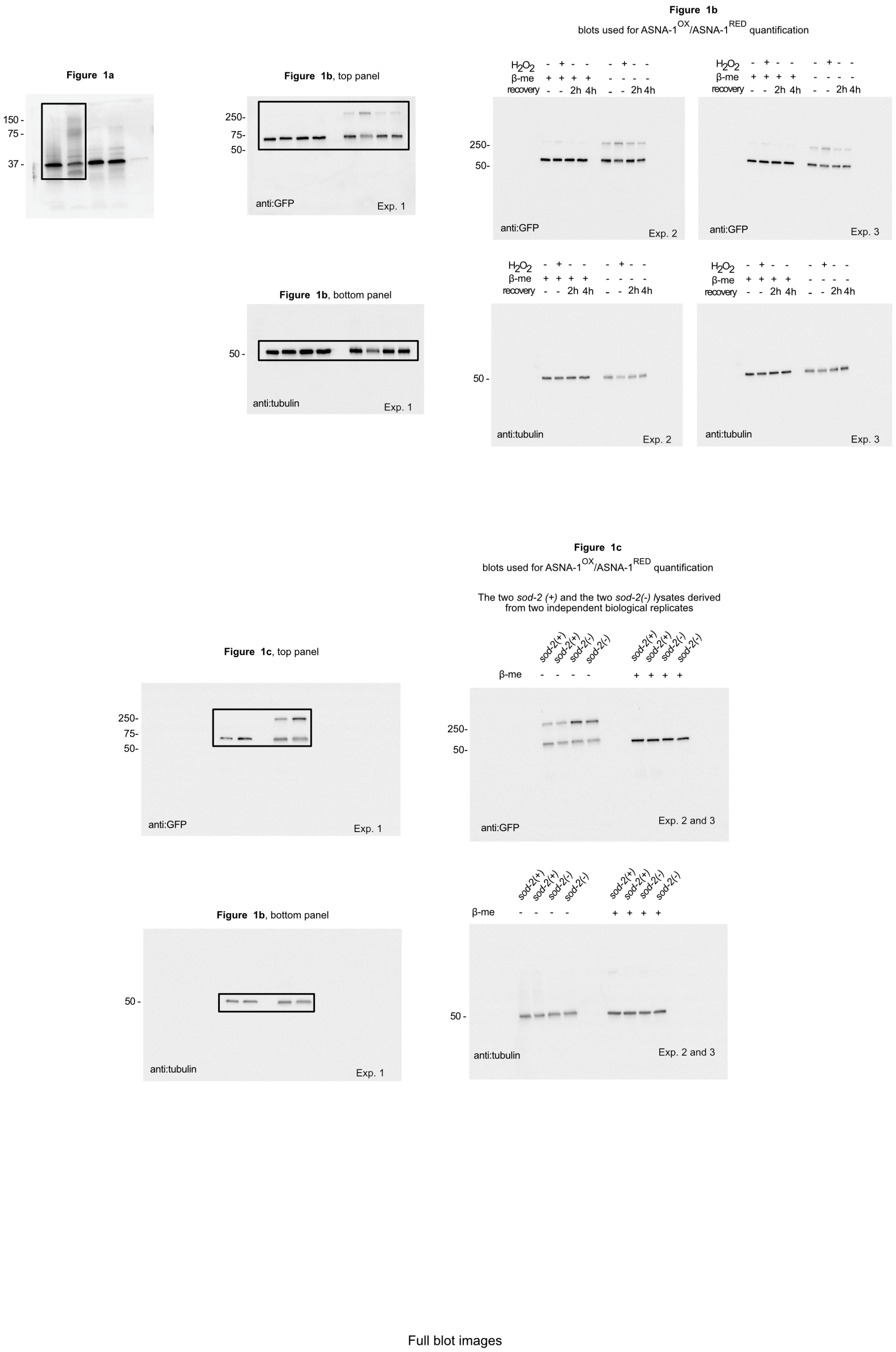

Figure S11. Full blot images.

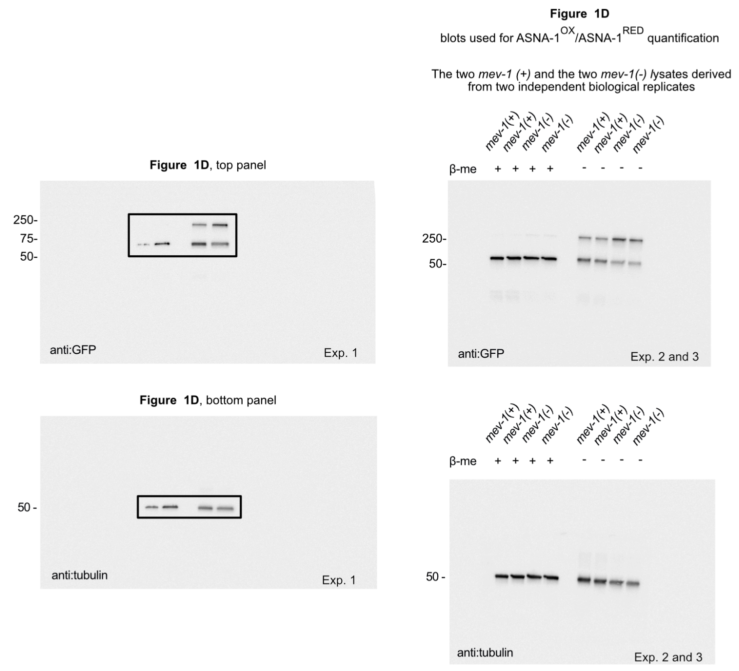

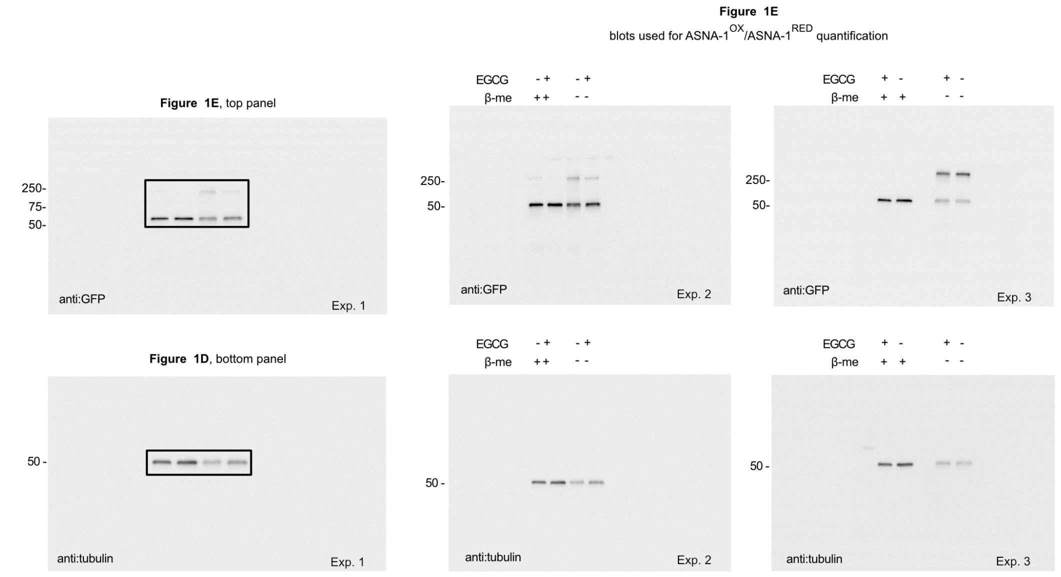

Full blot images

Figure S12. Full blot images.

Figure 3a, top panel

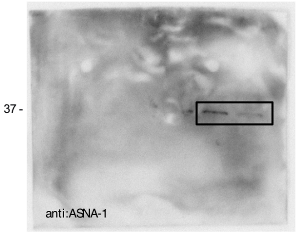

Figure 3a, bottom panel

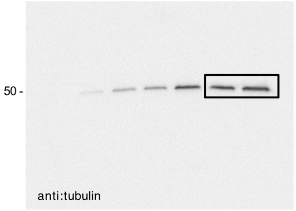

Full blot images

Figure S13. Full blot images.

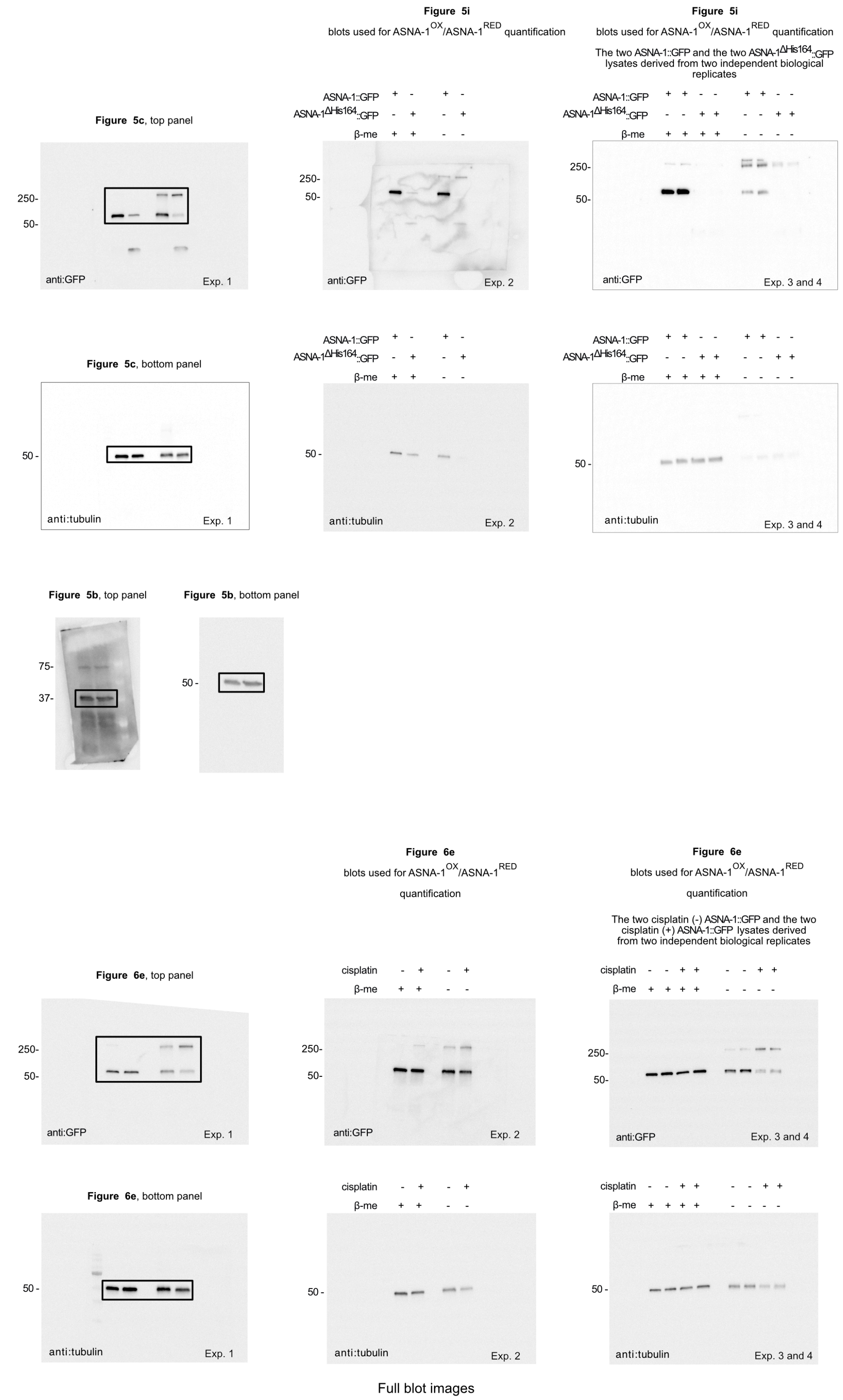

Figure S14. Full blot images.

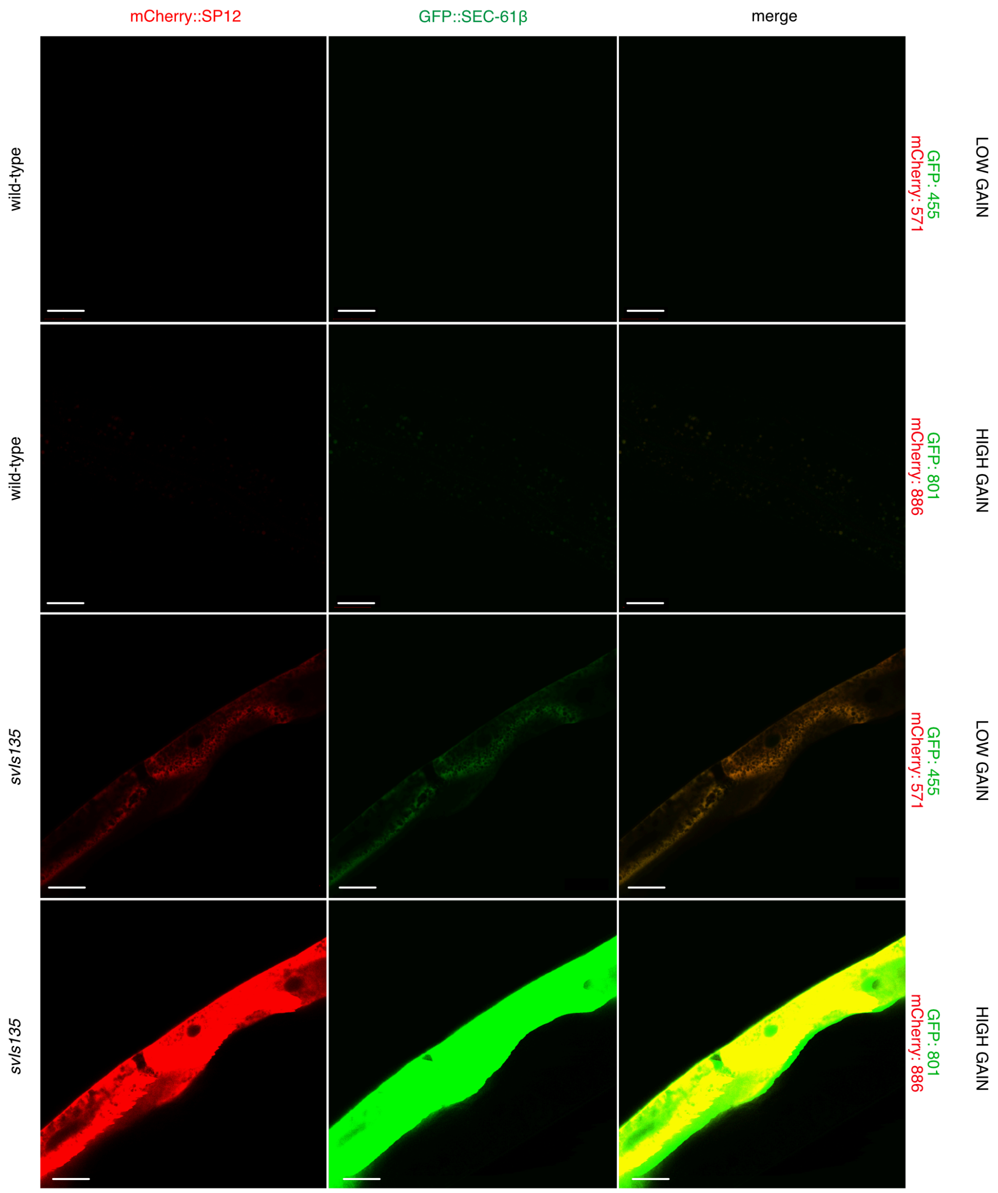

Figure S15. Representative confocal image of wild-type worms and transgenic worms (*svIs135*) adult animals co-expressing GFP::SEC-61β with mCherry::SP12 in intestinal int8 and int9 cells. Scale: 20 µm. To exclude possibility of autofluorescence signal from the intestine interfering with fluorescent signal seen in transgenic worms, we have used lower gain (455 for GFP and 571 for mCherry) to image transgenic (*svIs135*) animals. The same lower gain setting in wild-type animals did not give any autofluorescence signal. Higher gain setting (801 for GFP and 571 for mCherry) was used to show the first intestinal autofluorescence appearance in the wild-type worms. The same setting caused high oversaturation of the signal in the transgenic worms. Therefore, the low gain setting was used in all confocal pictures presented in this study.

SUPPLEMENTARY TABLES

Supplementary Table S2. *C. elegans* strains used in the study.

Strain name	Genetic background	Transgene	Notes	Reference
N2	Wild-type			
VB2906	*wrb-1(tm5938)* V /*nT1(qIs51)*		obtained from Mitani lab, 6xOC	this study
VB2907	*wrb-1(tm5532)* V /*nT1(qIs51)*			this study
GOT142	*asna-1(ok938)* III /*hT2(qIs48)*			[1]
GA184	*sod-2(gk257)* I		From CGC	
TK22	*mev-1(kn-1)* III		From CGC	
VB2760	*tom-1(ok285)* I		From CGC	
DA509	*unc-31(e928)* IV		From CGC	
PHX1544	*asna-1(syb1544)* III /*hT2(qIs48)*		*asna-1(ΔHis164)*	this study
GOT17	*svIs143*	*nhx-2p::mCherry::lgg-1*	genomic integration of *vkEx1093*	[2]
TJ356	*zIs356* IV	*daf-16p::daf-16a/b::GFP + rol-6(su1006)*	From CGC	
GOT123	*svIs135* X	*vha-6p::gfp::sec-61.B(Y38F2AR.9) + Pvha-6::mCherry::SP12*		this study
VB2721	*svEx917*	*vha-6p::gfp::cytb-5.1(C31E10.7) + Pvha-6::mCherry::SP12*		this study
VB1428	*svIs56*	*asna-1p::asna-1::GFP*		[1]
VB1605	*svIs69*	*daf-28p::daf-28::GFP + unc-4(+)*	3xOC	[1]
GOT120	*rawIs13*	*asna-1p::asna-1$^{C285S;C288S}$::GFP*		this study
COP224	*knuSi184* II; *unc-119(ed3)* III	*asna-1p::asna-1::HA::GFP::tbb-2 3'UTR*	inserted on chr II at the *ttTi5605* locus	this study
GOT352	*rawEx64*	*vha-6p::3xFlag::sec-61.B(Y38F2AR.9)::opsin + Pvha-6::mCherry::SP12*	2xOC	this study
GOT353	*rawIs19*	*vha-6p::3xFlag::sec-61.B(Y38F2AR.9)::opsin + Pvha-6::mCherry::SP12*	4xOC	this study
SJ4005	*zcIs4* V	*hsp-4::GFP*	From CGC	

EG8837	*unc-119(ed3)* III*;oxTi880* IV	*oxTi880[vha-6p::GFP::tbb-2 3'UTR + Cbr-unc-119(+)]*	From CGC	[3]
LD1171	*ldIs3*	*gcs-1p::GFP + rol-6(su1006)*	From CGC	
CL2166	*dvIs19* III	*(pAF15)gst-4p::GFP::NLS*	From CGC	
GOT195	*rawEx14*	*vha-6p::gfp::serp-1.1(F59F4.2) + Pvha-6::mCherry::SP12*		this study
GOT214	*rawEx21*	*Pvha-6::gfp::sec-61.B*$^{\Delta TMD}$ + *Pvha-6::mCherry::SP12*		this study
GOT163	*svEx591*	*asna-1p::asna-1*$^{\Delta His164}$*::GFP*		[4]
VB2699	*svIs136*	*vha-6p::mCherry::SP12*		this study

CGC: Caenorhabditis Genetics Center (https://cgc.umn.edu/)

SUPPLEMENTARY MATERIAL AND METHODS

Plasmids. The 1.8 kb intestine-specific *vha-6* promoter from pVB343ML[5] was cloned as a SacI/NheI fragment into pPD49.26 (Fire lab vector kit) and used as a backbone for all cloning with fluorescent markers. pVB637OB, pVB638OB:: GFP and mCherry were amplified without stop codons and inserted as KpnI/NheI fragments after the *vha-6* promoter. GFP insertion produced pVB637OB and mCherry insertion produced pVB638OB. Both plasmids contained the *unc-54* 3'UTR. pVB641OB: Genomic *spcs-1* (SP12) was amplified and inserted as a KpnI/KpnI fragment 3' to mCherry in pVB638OB. pVB643GK: The full-length *wrb-1* (Y50D4A.2) cDNA was cloned as a HinDIII fragment into L4440 to give pVB643GK and used to make *wrb-1* dsRNA using MegaScript T7 kit (ThermoScientific). pVB644OB: The full-length error free cDNA of *wrb-1* was synthesized as a KpnI/KpnI fragment (GenScript Inc., NJ, USA) and cloned 3' to the GFP coding region in pVB637OB. GFP::SEC-61 β: The open reading frame of Y38F2AR.9 *(sec-61.B)* was amplified and inserted as a KpnI fragment 3' to the coding region of GFP in pVB637OB to yield pVB639OB. GFP::CYTB-5.1 The open reading frame of *cytb-5.1* (Cytochrome B5) was amplified and inserted as a KpnI fragment 3' to the GFP coding region in pVB637OB to yield pVB640OB. GFP::SERP-1.1: The genomic region of the TAP *serp-1.1* lacking the initiator methionine was amplified with Q5 polymerase and inserted downstream of and in frame with GFP in pVB637OB to generate the plasmid pDR28 using Gibson assembly technology (New England Biolabs). The genomic clone of the worm sec-61β lacking the TMD was synthesized by Genscript (Piscataway, USA) and the clone lacking the initiator methionine was inserted in frame downstream of GFP in pVB637OB using Gibson ligation technology to yield pGK208. 3xFlag::SEC61β::opsin tag was synthesized as a 430nt fragment (Genscript) and cloned downstream of the *vha-6* promoter to replace GFP in pVB637OB using Gibson ligation technology (New England Biolabs) to produce pGK213 (vha-6p::3xFlag::SEC61β::opsin tag).

Transmission electron microscopy (TEM). Worms were washed 3x in M9. The anterior portion of the head was cut off in fixative solution, 2.5 % (v/v) glutaraldehyde in 0.1 M cacodylate. Samples were incubated overnight in fixative at 4 °C, washed 3x in fixative solution, treated with 1 % (v/v) osmium tetroxide for 1 h and washed 2x in distilled water. Dehydration with 50, 70, 95, and 100 % ethanol was followed by infiltration and embedment in Spurr's resin. Using a DiATOME diamond knife on a Leica EM UC7, sections (70 nm) were mounted on copper grids. The grids were treated with 5 % uranyl acetate in water for 20 minutes, followed by Sato's lead staining for 5 minutes. Sections were examined with a Jeol

1230 transmission electron microscope; images were captured using a Gatan MSC 600CW camera.

SUPPLEMENTARY REFERENCES

1. Kao, G. *et al.* ASNA-1 Positively Regulates Insulin Secretion in C. elegans and Mammalian Cells. *Cell* **128**, 577–587 (2007).

2. Gosai, S. J. *et al.* Automated high-content live animal drug screening using C. elegans expressing the aggregation prone serpin α1-antitrypsin Z. *PLoS One* **5**, e14460 (2010).

3. Frokjaer-Jensen, C. *et al.* Random and targeted transgene insertion in C. elegans using a modified Mosl transposon. *Nat. Methods* **11**, 529–534 (2014).

4. Hemmingsson, O., Kao, G., Still, M. & Naredi, P. ASNA-1 activity modulates sensitivity to cisplatin. *Cancer Res.* **70**, 10321–10328 (2010).

5. Larsen, M. K., Tuck, S., Færgeman, N. J. & Knudsen, J. MAA-1, a Novel Acyl-CoA–binding Protein Involved in Endosomal Vesicle Transport in Caenorhabditis elegans. *Mol. Biol. Cell* **17**, 4318–4329 (2007).
